# Host phylogeny and life history stage shape the gut microbiome in dwarf (*Kogia sima*) and pygmy (*Kogia breviceps*) sperm whales

**DOI:** 10.1038/s41598-020-72032-4

**Published:** 2020-09-16

**Authors:** Elizabeth R. Denison, Ryan G. Rhodes, William A. McLellan, D. Ann Pabst, Patrick M. Erwin

**Affiliations:** Department of Biology and Marine Biology, Center for Marine Science, University of North Carolina Wilmington, Wilmington, NC 28409 USA

**Keywords:** Marine biology, Microbiome, Symbiosis

## Abstract

Gut microbiomes perform crucial roles in host health and development, but few studies have explored cetacean microbiomes especially deep divers. We characterized the gut microbiomes of stranded dwarf (*Kogia sima*) and pygmy (*K. breviceps*) sperm whales to examine the effects of phylogeny and life stage on microbiome composition and diversity. 16S rRNA gene sequence analysis revealed diverse gut communities (averaging 674 OTUs) dominated by a few symbiont taxa (25 OTUs accounted for 64% of total relative abundance). Both phylogeny and life stage shaped community composition and diversity, with species-specific microbiome differences present early in life. Further analysis showed evidence of microbiome convergence with host maturity, albeit through different processes: symbiont ‘accumulation’ in *K. sima* and ‘winnowing’ in *K. breviceps*, indicating different methods of community assembly during host development. Furthermore, culture-based analyses yielded 116 pure cultures matching 25 OTUs, including one isolate positive for chitin utilization. Our findings indicate that kogiid gut microbiomes are highly diverse and species-specific, undergo significant shifts with host development, and can be cultivated on specialized media under anaerobic conditions. These results enhance our understanding of the kogiid gut microbiome and may provide useful information for symbiont assessment in host health.

## Introduction

Host-associated microbes are established members of nearly every living organism^1^ and can be essential for health homeostasis^2, 3^. The intestinal microbiome, in particular, plays a critical role in mammalian health and development^4^. Intestinal symbionts are hypothesized to contribute to three fundamental properties that benefit the host and perhaps encourage host-specificity: (1) nutrient acquisition (i.e. digesting otherwise un-digestible material), (2) trophic processes (immune system development and epithelial cell regulation), and (3) protection against pathogens via competitive exclusion (i.e. colonization resistance)^5, 6^. It is speculated that core intestinal symbionts are gut-specific, possessing genes essential for survival in the gut environment^7^. Phylogeny strongly influences the composition and diversity of mammalian microbiomes, with an individual’s microbial profile remarkably similar to other individuals of the same species^8, 9^. Thus, regardless of age, sex or geographic location, a given host species contains a shared set of symbionts, or symbiont functions, termed a ‘core’ microbiome^10–12^. Within host species, individual variation in microbiome composition also occurs, including individuals harboring unique symbiont taxa^11^, that result from factors such as diet, environment, and host physiology, which collectively shape microbiomes as unique as a fingerprint.

For marine mammals, microbiome characterization represents a promising method to monitor individual, population, and ecosystem health during a time when many species are under threat. As apex predators with relatively slow reproductive rates, marine mammals are particularly susceptible to environmental and anthropogenic stressors (e.g. pollutants, ocean-borne plastics, and commercial fishing practices)^13^, with documented evidence of both direct (e.g. fisheries) and indirect (e.g. pollution) anthropogenic impacts threatening marine mammals globally^14^. However, relatively few investigations have examined the microbiomes of marine mammals and their roles in host health^9^. The overall lack of comprehensive microbiome data, coupled with increasing ecosystem health concerns as well as technological advancements, has expedited efforts to elucidate the marine mammal microbiome and its role in host health^15^.

Investigations into the gut microbiomes of baleen whales^16^, dugongs, manatees, and a variety of pinnipeds and dolphins have been conducted^9^. From this pool of species, the observed trends in microbiome composition mirror those in terrestrial mammals, namely the strong influence of phylogeny on community assembly and the species-specific nature of the mammalian microbiome^17^. Marine mammals have unique microbiomes that are distinct from those of terrestrial relatives^17^, dietary fish^10^, and their surrounding environment (i.e. seawater)^10^. In fact, seawater profiles from different oceans are more similar to one another than are profiles from the same body site in different marine mammal species^10^. While environmental variation may cause shifts in microbiome composition, these data suggest that phylogeny has a prevailing influence over the microbiome.

Despite progress in characterizing the microbiomes of selected marine mammal species^9, 15^, little information is available for odontocete (toothed) cetaceans. Deep-diving kogiid whales are of particular interest as they are the second most commonly stranded marine mammal in the southeastern United States^18^. The genus *Kogia* consists of two extant species, *Kogia sima* (dwarf sperm whale) and *K*. *breviceps* (pygmy sperm whale). There is significant dietary overlap between the two species, although there is evidence their diets differ slightly, possibly due to niche partitioning (i.e. water temperature and depth)^18^. Kogiid offshore distribution and behavioral tendencies (e.g. inconspicuous surfacing behavior and extended dive times^19^) render noninvasive, systematic sampling of free-ranging individuals unlikely. Moreover, stranded *Kogia* do not survive long in rehabilitation and are not captured in the wild^20^. Accordingly, the majority of studies on kogiid whales are opportunistic in nature and utilize samples collected from stranded debilitated or dead animals. Strandings provide essential insights into their preferred habitat, dietary choices, and, recently, their microbial symbionts^13, 18, 21, 22^. Characterizing the kogiid intestinal microbiome may elucidate its role in kogiid health homeostasis and help better understand their relatively frequent strandings and clinical challenges during rehabilitation.

Although kogiid whales and their microbial symbionts remain largely unexplored, the gut microbiome of adult kogiid whales has recently been investigated, indicating a high degree of host-specificity of microbiomes within the genus^22^. *K. sima* and *K. breviceps* exhibit unique gut profiles both in diversity and community composition^22^. Kogiid microbiome communities clustered more closely with baleen whales than other toothed whales, though some dominant phyla in kogiid whales were also rare in baleen whales, further distinguishing the kogiid microbiome as its own entity^22^. Overlap between kogiid gut microbes and those of other cetacean species may reflect ancient, functional adaptations to life at sea. A more comprehensive understanding of gut microbiome development and maintenance is required to elucidate its role in kogiid health. Thorough characterizations of the kogiid microbiome and its most dominant core members are also needed to be successfully applied to health monitoring and conservation efforts. Two specific gaps in knowledge are: (1) the effects of life stage on the gut microbiome, and (2) the functional roles of individual taxa within the host.

In this study, we used stranded kogiids from the mid-Atlantic United States to apply both sequence and culture-based methods to characterize the kogiid gut microbiome. Our objectives were to characterize the gut microbiome in juvenile kogiids and compare the composition and diversity with those of adults using culture-independent (molecular) techniques. We also aimed to couple culture-based and sequence-based approaches in kogiid whales to link overall microbiome composition and symbiont function, a two-step process. First, dominant symbionts must be isolated in pure culture. Second, viable cultures must be experimentally tested for specific functional attributes. While the primary focus herein was the former step, we also conducted a chitin utilization assay as proof-of-concept for the latter. Chitin degradation is a potentially beneficial symbiont function in the kogiid gut, since chitin is a structural feature of kogiid prey (cephalopods with chitinous beaks and pens)^18^, is difficult for mammals to digest and represents a nutrient source for some microbes^23^. Work in humans has revealed advantages to pairing traditional culturing methods with sequence-based approaches to provide in-depth characterizations of gut microbiome symbionts^23^. There have been very few attempts to culture cetacean gut symbionts, but culturing may prove useful in understanding the gut microbiome during health and disease^24, 25^. We hypothesized that (1) kogiid gut microbiomes will exhibit differences in composition and diversity based on host life stage, and (2) dominant kogiid gut symbionts can be isolated in pure culture by optimizing growth conditions for anaerobic spore-formers^26^.

## Results

### Phylum-level composition

**Figure 1:**
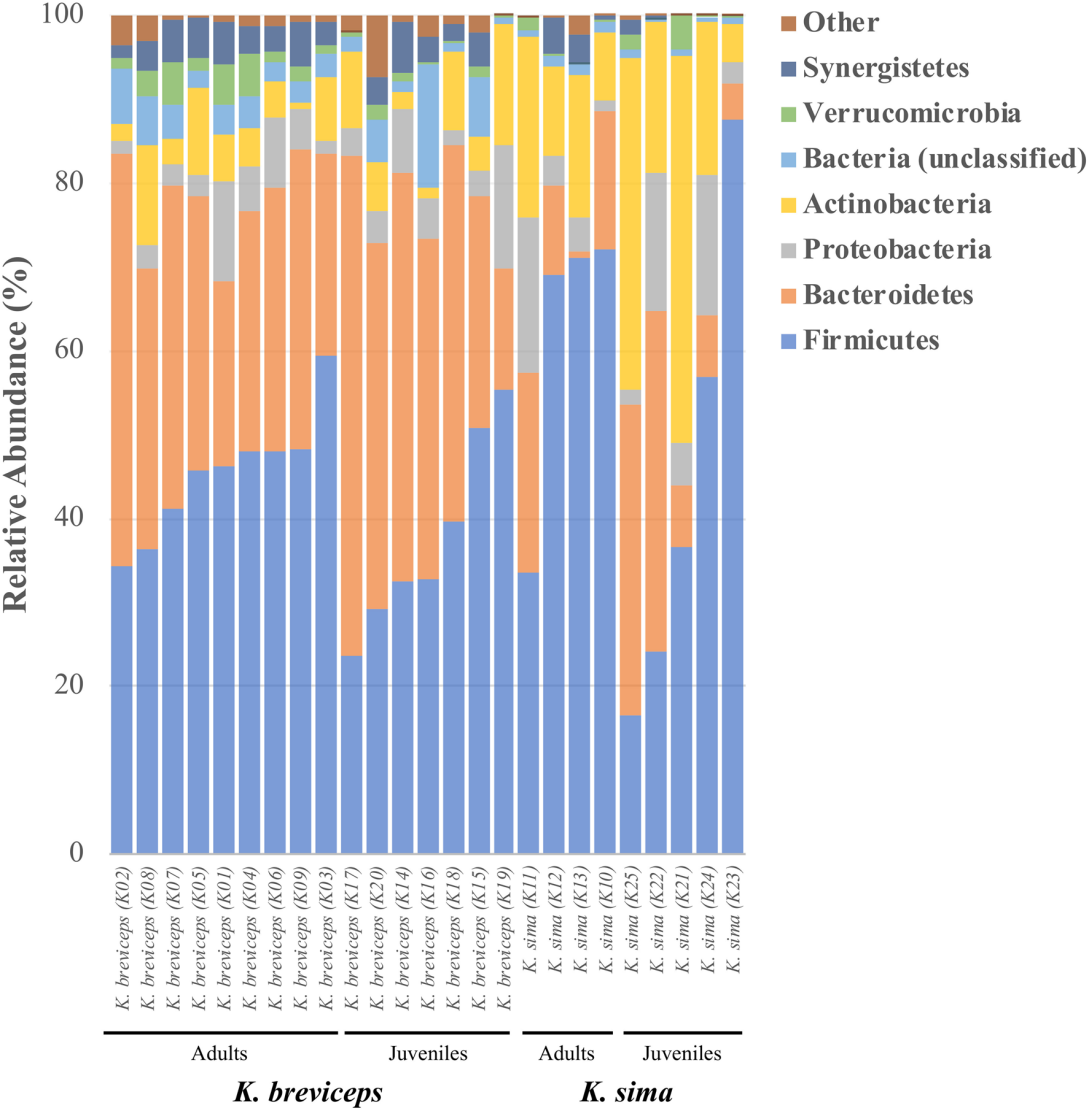
Phylum-level composition of gut microbiomes in adult and juvenile *K. sima* and *K. breviceps*. Relative abundance shown as percentage. Rare phyla (Other) consist of Lentisphaerae, Tenericutes, Euryarchaeota, Cyanobacteria, Spirochaetes, and Fusobacteria*.*

A total of 5,944 OTUs were recovered from all 25 individual kogiids, encompassing one archaeal phylum and 11 bacterial phyla (Fig. 1). Firmicutes and Bacteroidetes dominated the kogiid gut microbiome, together accounting for ~ 75% of the total gut communities and ~ 66% of all recovered OTUs (Table S1). Actinobacteria, Proteobacteria, Synergistetes, and Verrucomicrobia were also relatively common phyla, in addition to six rare (< 1% relative abundance) phyla (Table S1). Abundant phyla owed their dominance to a small fraction of the total OTUs belonging to each lineage: five OTUs (< 1% of total Bacteroidetes OTUs) constituted ~ 85% of all Bacteroidetes, and only two OTUs (< 1% of total Actinobacteria OTUs) accounted for ~ 53% of all Actinobacteria symbionts.

Phylum-level differences in composition were observed between kogiid species, but not between life history stages. Actinobacteria, Bacteroidetes, and Synergistetes exhibited significant differences in their relative abundances between *K. sima* and *K. breviceps* (all life history stages), but no significant phylum-level differentiation occurred between juvenile and adult gut microbiomes (both hosts, Table 1). Similarly, comparisons of phylum-level differences within each host species revealed no significant differences across life history stages for *K. sima* and only one differentially abundant phylum in *K. breviceps* (Verrucomicrobia, Table S2).

**Table 1:** Comparisons of relative abundance (± SD) of bacterial and archaeal phyla in kogiid gut microbiomes between life history stage (juvenile and adult) and host species (*K. sima* and *K. breviceps*).

Phylum	Life history stage			Host species		
	Juvenile	Adult	*p*	*K. sima*	*K. breviceps*	*p*
Firmicutes	40.51 ± 19.56	50.24 ± 13.58	0.159	51.95 ± 24.9	41.69 ± 10.21	0.278
Bacteroidetes	31.31 ± 18.65	26.76 ± 12.59	0.479	16.49 ± 14.34	36.88 ± 11.42	0.001*
Actinobacteria	14.41 ± 14.58	8.28 ± 6.03	0.196	20.47 ± 13.94	6.06 ± 4.25	0.015*
Proteobacteria	6.81 ± 5.70	5.30 ± 5.02	0.491	7.78 ± 7.16	4.57 ± 3.47	0.311
Synergistetes	1.77 ± 2.01	3.29 ± 1.73	0.053	1.22 ± 1.69	3.21 ± 1.78	0.008*
Verrucomicrobia	0.98 ± 1.19	2.04 ± 1.86	0.106	0.89 ± 1.37	1.69 ± 1.55	0.143
Lentisphaerae	0.73 ± 1.47	0.29 ± 0.30	0.329	0.07 ± 0.11	0.78 ± 1.29	0.049
Euryarchaeota	0.26 ± 0.55	0.11 ± 0.20	0.396	0.01 ± 0.02	0.29 ± 0.50	0.048
Tenericutes	0.25 ± 0.42	0.68 ± 0.78	0.100	0.26 ± 0.69	0.60 ± 0.65	0.228
Spirochaetes	0.05 ± 0.14	0.02 ± 0.02	0.553	0.01 ± 0.01	0.05 ± 0.12	0.137
Cyanobacteria	0.04 ± 0.11	0.03 ± 0.06	0.700	0.04 ± 0.07	0.03 ± 0.10	0.886
Fusobacteria	0.01 ± 0.03	0.03 ± 0.08	0.433	0.01 ± 0.00	0.04 ± 0.08	0.130

Asterisks (*) indicated phyla exhibiting significant differences in relative abundance between hosts following B–Y corrections. All phyla belong to the domain Bacteria, expect Euryarchaeota from the domain Archaea.

### OTU-level composition

Fifty OTUs comprised the kogiid core gut microbiomes (i.e. OTUs present in every sampled individual), representing six bacterial phyla with over half of the core OTUs belonging to the phylum Firmicutes (Table S3). Together, core OTUs accounted for only ~ 10% of the total number of OTUs, but ~ 75% of the total number of recovered sequences. The trend of a small fraction of OTUs dominating a large portion of the total community was also observed when identifying core communities in juvenile and adult kogiids. Adult kogiids harbored the largest core community, consisting of 136 OTUs that comprised ~ 93% of total community, but only ~ 1% of the total number of OTUs present in adults (Table S3). Juvenile kogiids shared fewer OTUs, their core consisting of 54 OTUs that comprised ~ 78% of the total community yet ~ 1% of the total number of OTUs detected in juveniles (Table S3). Between juvenile and adult kogiids, 50 of the 54 core juvenile taxa were present in the adult core communities. The 4 core juvenile taxa not detected in adult communities corresponded to 2 OTUs affiliated with the phylum Bacteroidetes (OTU00089, OTU00226) and 2 OTUs affiliated with the phylum Firmicutes (OTU00080 genus *Ruminococcus*, OTU00137 order Clostridia; Table S3). Overall, the core symbionts dominated kogiid gut microbiomes, with 24 of the 25 most abundant OTUs present in the kogiid core (Table S3).

The 25 most common OTUs were the primary drivers of community divergence and exhibited differential abundances between life history stages and host species. Together, these 25 abundant OTUs accounted for > 50% of the dissimilarity between juveniles and adults (in both host species, Table S4) and > 50% of the dissimilarity between *K. sima* and *K. breviceps* (in both life stages, Table 2). Comparing across host species, juvenile kogiids exhibited more differentially abundant taxa (n = 8) compared to adults (n = 4), with greater contributions to community dissimilarity (23% in juveniles, 12% in adults, Table 2). Comparing within host species, *K. breviceps* exhibited greater microbiome shifts across life stages compared to *K. sima*, with four differentially abundant OTUs contributing 6.6% to community dissimilarity (compared to 1 OTU contributing 3.7% in *K. sima*, Table S4). Thus, at the OTU level, host species had a stronger effect on microbiome composition than life stage.

**Table 2:** The 25 most common OTUs and their relative abundance (± SD) in juvenile and adult kogiid whales.

OTU	Lowest taxonomy	Adults			Juveniles		
		*K. sima*	*K. breviceps*	% Contrib	*K. sima*	*K. breviceps*	% Contrib
00001	p_Bacteroidetes	**0.79 ± 0.51**	**10.68 ± 7.36**	**7.04**	**1.24 ± 2.01**	**16.71 ± 12.34**	**9.04**
00002	p_Bacteroidetes	8.41 ± 6.41	12.07 ± 2.2	3.86	8.46 ± 9.18	7.83 ± 6.47	4.83
00003	f_Peptostreptococcaceae	0.89 ± 0.62	8.74 ± 6.27	5.71	**0.09 ± 0.03**	**7.51 ± 8.11**	**4.33**
00004	f_Peptostreptococcaceae	5.89 ± 6.22	1.43 ± 1.85	3.91	3.86 ± 8.23	0.06 ± 0.03	2.23
00005	g_*Adlercreutzia*	3.33 ± 1.97	0.37 ± 0.15	2.12	11.03 ± 17.15	0.61 ± 0.78	6.36
00006	*Clostridium perfringens*	8.83 ± 11	1.66 ± 2.65	6.18	0.25 ± 0.55	0.51 ± 1.25	0.39
00007	f_Mogibacteriaceae	1.56 ± 1.83	5.04 ± 1.46	2.60	**0.07 ± 0.03**	**3.71 ± 2.68**	**2.13**
00008	o_Clostridia	6.82 ± 2.47	3.25 ± 3.91	3.56	0.81 ± 1.08	0.66 ± 0.45	0.47
00009	p_Bacteroidetes	0.38 ± 0.41	1.41 ± 1.09	0.82	**0.71 ± 1.02**	**3.76 ± 4.42**	**1.97**
00010	f_Synergistaceae	**0.14 ± 0.16**	**3.6 ± 1.28**	**2.46**	**0.30 ± 0.62**	**2.52 ± 2.02**	**1.38**
00011	g_*Mycobacterium*	1.28 ± 1.11	3.86 ± 3.77	2.39	2.43 ± 4.33	3.23 ± 3.32	2.22
00012	o_Bacteroidales	**0.08 ± 0.06**	**3.31 ± 1.99**	**2.30**	**0.19 ± 0.30**	**4.57 ± 3.68**	**2.57**
00013	o_Clostridia	3.9 ± 3.74	1.32 ± 1.31	2.34	4.75 ± 9.35	0.99 ± 1.66	2.88
00014	o_Clostridia	**0.21 ± 0.12**	**1.57 ± 1.57**	**0.97**	**0.13 ± 0.15**	**3.01 ± 2.53**	**1.69**
00015	p_Bacteroidetes	1.49 ± 2.67	1.91 ± 2.26	1.62	3.07 ± 6.8	1.41 ± 1.82	2.25
00016	f_Enterobacteriaceae	0.42 ± 0.46	0.68 ± 1.3	0.56	3.91 ± 6.88	0.06 ± 0.06	2.28
00017	g_*Oscillospira*	2.36 ± 2.91	1.43 ± 0.62	1.33	0.19 ± 0.37	0.47 ± 0.38	0.26
00018	g_*Oscillospira*	0.78 ± 0.52	2.54 ± 1.41	1.26	0.42 ± 0.62	0.52 ± 0.63	0.34
00019	*Campylobacter fetus*	0.05 ± 0.01	2.56 ± 3.38	1.79	0.00 ± 0.00	0.39 ± 1.02	0.23
00020	f_RFP12	0.33 ± 0.5	2.48 ± 1.72	1.56	0.34 ± 0.74	0.71 ± 0.60	0.44
00021	f_Lachnospira	0.27 ± 0.07	1.48 ± 0.62	0.85	**0.05 ± 0.02**	**2.82 ± 3.22**	**1.61**
00022	g_*Desulfovibrio*	1.34 ± 1.67	0.46 ± 0.51	0.85	3.65 ± 7.00	2.88 ± 5.02	2.82
00025	f_Coriobacteriaceae	1.99 ± 1.48	0.06 ± 0.03	1.37	3.37 ± 6.84	0.01 ± 0.01	1.96
00030	f_Coriobacteriaceae	1.91 ± 2.74	0.05 ± 0.03	1.32	4.22 ± 8.77	0.02 ± 0.01	2.45
00037	f_Ruminococcaceae	0.06 ± 0.06	0.04 ± 0.06	0.04	5.17 ± 9.45	0.45 ± 0.99	3.01

Percent contributions to dissimilarity between host species (% Contrib.) determined by SIMPER analysis. Values in bold represent differentially abundant symbionts between kogiid hosts (*p* < 0.05 for MetaStats and LefSe analyses).

### Microbiome similarity and diversity

Community-level analysis of the gut microbiomes revealed sharp distinctions between host microbiomes. *K. sima* and *K. breviceps* hosted significantly distinct gut microbiomes based on OTU relative abundance (Table 3, PERMANOVA*, p* < 0.001). Comparisons of juvenile and adult whales further revealed significant shifts in microbiome similarity across life stages (PERMANOVA, *p* < 0.001). Differences between life stage remained when considering an individual host species, with strong distinctions between juvenile and adult gut microbiomes in *K. sima* (PERMANOVA, *p* < 0.004) and *K. breviceps* (PERMANOVA, *p* < 0.002). No significant differences were detected in community similarity based on sex (ANOSIM, *p* = 0.985) or carcass condition at examination (ANOSIM, *p* = 0.260). Significant differences in microbiome similarity were also detected between hosts and life history stages when considering symbiont membership (OTU presence-absence, PERMANOVA *p* < 0.015), indicating differences across hosts and ontogeny are driven by both the relative abundance of taxa and by symbiont membership (Table 3).

**Table 3:** Pairwise statistical comparisons of microbiome similarity based on OTU-dependent (Bray Curtis) and OTU-independent (UniFrac) metrics of relative abundance (Rel. Abund., Weighted) and presence-absence (Presence-Abs., Unweighted) data.

Pairwise comparison	Bray–Curtis similarity				UniFrac distance			
	Rel. Abund		Presence-Abs		Weighted		Unweighted	
	*t*	*P*	*t*	*P*	*t*	*P*	*t*	*P*
***K. sima versus K. breviceps***								
Both life history stages	2.245	< 0.001*	1.532	< 0.001*	2.727	< 0.001*	1.278	0.002*
Adults	1.999	0.001*	1.279	0.002*	2.581	0.002*	1.157	0.001*
Juveniles	1.654	0.003*	1.409	0.002*	1.723	0.007*	1.178	< 0.001*
**Adults versus Juveniles**								
Both host species	1.886	< 0.001*	1.832	< 0.001*	1.456	0.035*	1.439	< 0.001*
*K. breviceps*	1.730	< 0.001*	1.710	< 0.001*	1.202	0.151	1.352	0.001*
*K. sima*	1.295	0.032*	1.380	0.014*	1.053	0.341	1.175	0.007*

Asterisks (*) indicate significant differences.

**Figure 2:**
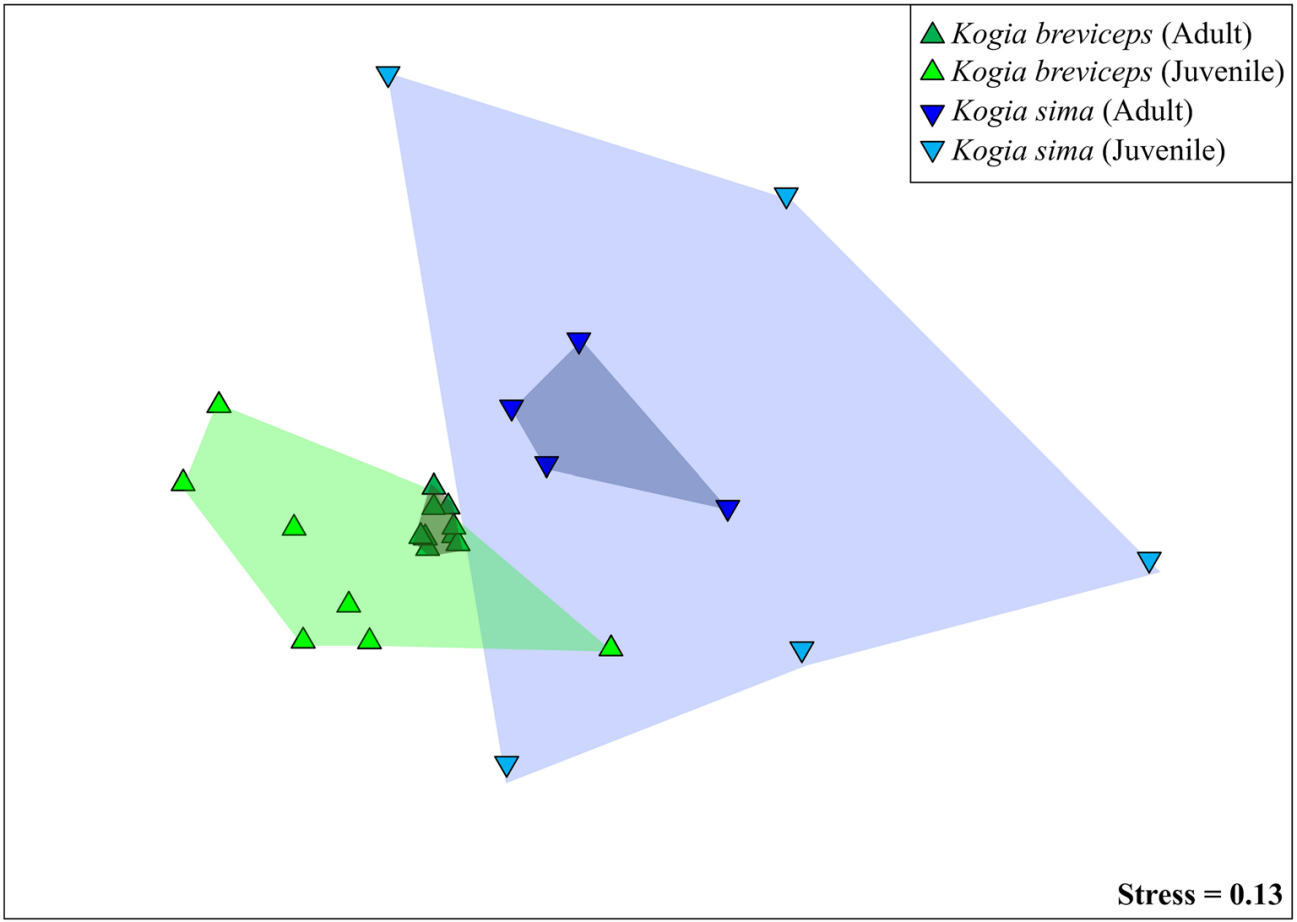
Non-metric multidimensional scaling (NMDS) plot of the gut microbiome in juvenile and adult *K. sima* (green shading) and *K. breviceps* (blue shading). Ordination is based on Bray–Curtis similarity. Gut microbiomes differed significantly (PERMANOVA, *p* < 0.05) across host species (*K. sima* vs. *K. breviceps*) and life stage (juvenile vs. adult).

Non-metric multidimensional scaling analysis (NMDS) indicated that kogiid gut microbiomes converge with host maturity based on OTU-dependent (Fig. 2) and OTU-independent (Figs. S1, S2) metrics. Indeed, significant differences in dispersion were detected between life stages and species (Table S5, PERMDISP, *p* < 0.01), indicating microbiome differences across these factors may also be attributed to heterogenous dispersion among groups. NMDS ordination plots highlight a more profound impact of this phenomenon between life stages (similar centroids) than species (distinct centroids), with adult gut microbiomes more similar to one another than were those of juveniles (55.96% versus 75.59% average dissimilarity). Further, within each host species, kogiid gut microbiomes became more similar with host maturity (*K. sima* juveniles = 12.48%, *K. sima* adults = 33.33%; *K. breviceps* juveniles = 40.76%, *K. breviceps* adults = 60.11%). While communities became more similar with age, it is important to note that both adult and juvenile community profiles remained species-specific (*p* < 0.008, Table 3). The two juvenile *K. breviceps* from cow-calf pairs did not exhibit greater similarity to their mothers than unrelated juvenile and adult *K. breviceps* (Fig. S3). While the cow-calf pairs did not group together on a cluster dendrogram, the mothers did cluster together (Fig. S3). The mothers were ~ 68% similar based on OTU relative abundance, while adult *K. breviceps* on average were ~ 60% similar.

**Figure 3:**
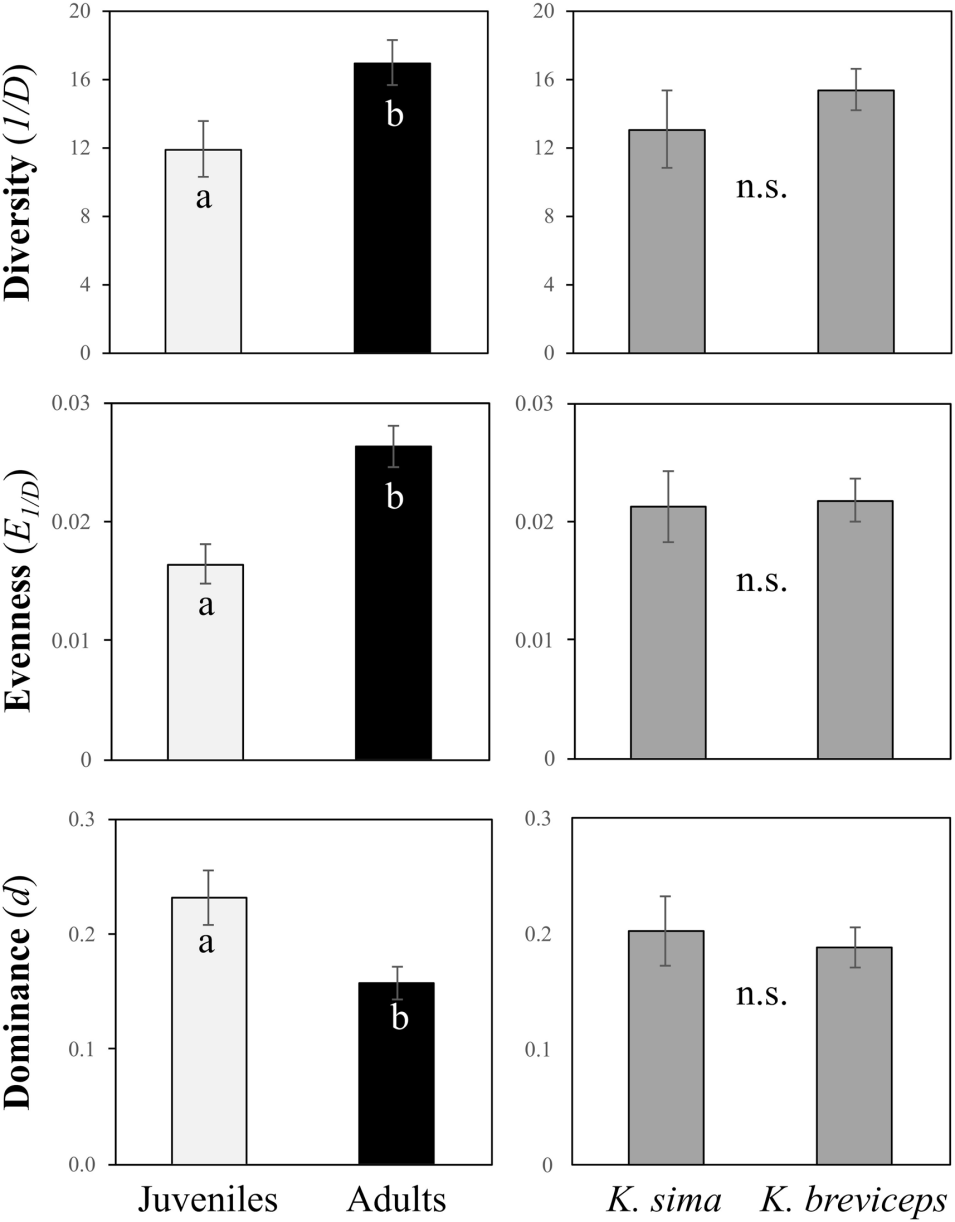
Diversity, evenness and dominance metrics of gut microbiomes in juvenile and adult *K. sima* and *K. breviceps*. Different letters indicate significantly different means (ANOVA, *p* < 0.05). Error bars represent ± 1 SE; n.s., not significant.

**Figure 4:**
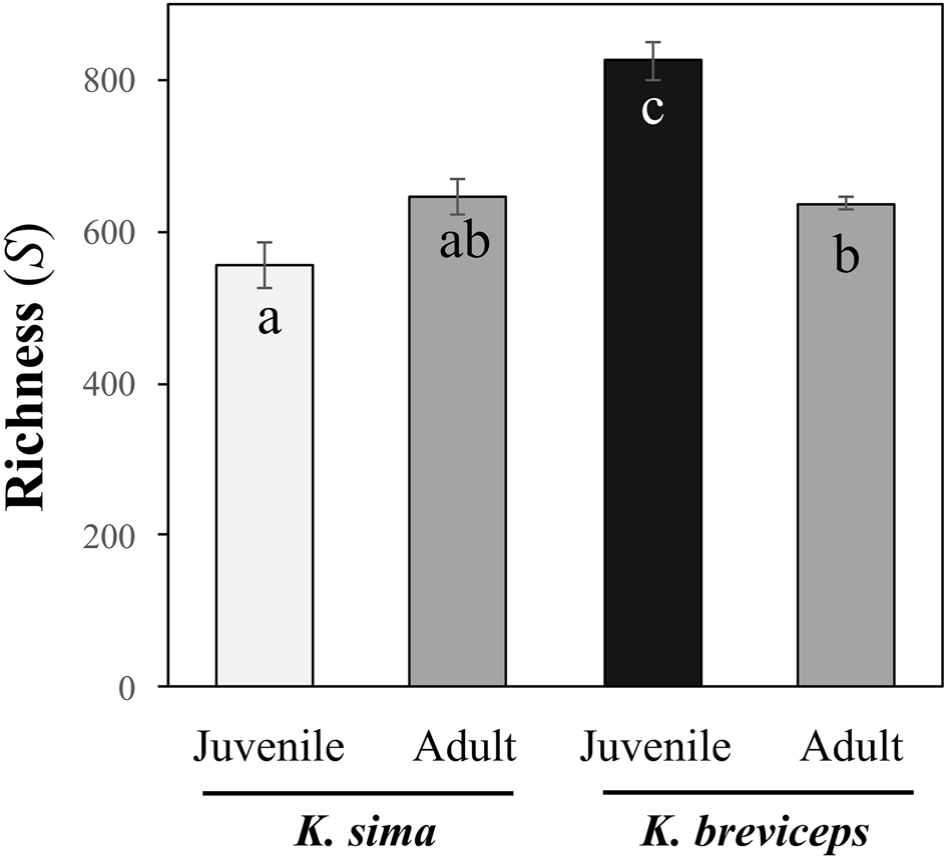
Comparison of mean OTU richness (S) in the gut microbiome of juvenile and adult *K. sima* and *K. breviceps*. Bars not connected by the same letter are significantly different (post hoc Tukey’s HSD). Error bars represent ± 1 SE.

Kogiid hosts exhibited high OTU-level diversity (Table S6), with the diversity (*1*/*D*), evenness (*E*_*1*/*D*_), and dominance (*d*) of gut microbial communities differing significantly between life stages (ANOVA, *p* < 0.005), but not between species (ANOVA, *p* > 0.050, Fig. 3). A significant interaction between host species and life history stage (ANOVA, *p* < 0.010) was detected for species richness (*S*), indicating variation in OTU richness between hosts was dependent upon life stage. Indeed, while juveniles exhibited higher OTU richness than adults when grouping data from both host species, these trends were driven by the high richness in juvenile *K. breviceps* (824 ± 24 SE, Fig. 4). Accordingly, symbiont richness was significantly higher in juvenile *K. breviceps* compared to juvenile *K. sima* and adult *K. breviceps* (post hoc Tukey’s HSD, *p* < 0.05, Fig. 4). No significant difference in richness was observed between adults, suggesting that symbiont richness stabilizes with host maturity across both species (post hoc Tukey’s HSD *p* > 0.05). Interestingly, while symbiont richness declined with age in *K. breviceps*, it appeared to increase with age in *K. sima*, although not significantly, illustrating differences in how the gut microbiome develops with host maturity in these two closely related species of cetaceans.

### Cultured representatives

A total of 116 isolates were cultured from kogiid fecal samples, representing three bacterial phyla: Firmicutes, Bacteroidetes, and Proteobacteria (Table 4). The majority of cultured symbionts were affiliated with the phylum Firmicutes, followed by Bacteroidetes and Proteobacteria. The 116 isolates matched to 25 OTUs from the culture-independent microbiome dataset with a threshold of > 97% similarity. Two cultured OTUs were of particular interest for downstream characterization: OTU00004 and OTU00006. These OTUs were identical matches (100% sequence identity) to dominant symbiont OTUs in the microbiome dataset, core members of each kogiid host, and among the 25 most dominant symbionts (Tables 2, Table S3). Additionally, OTU00004 was differentially abundant between juvenile and adult *K. breviceps* hosts (Table S4). When classified with the Greengenes database, OTU00004 was affiliated with the family Peptostreptococcaceae, and was further classified as *Clostridium sordellii* with the National Centre for Biotechnology Information (NCBI) non-redundant database. OTU00006 was identified as *Clostridium perfringens*. These data highlight the ability of complex culture media and anaerobic conditions to isolate dominant cetacean gut symbionts in pure culture.

**Table 4:** Summary counts and taxonomy of cultured isolates from *K. sima* and *K. breviceps* fecal samples.

OTU	Total	Juvenile	Adult	Phylum (lowest taxonomy)
00004	18	11	7	Firmicutes (f_Peptostreptococcaceae)
00006	27	10	17	Firmicutes (*Clostridium perfringens*)
00016	1	1	0	Proteobacteria (f_Enterobacteriaceae)
00032	2	1	1	Firmicutes (*Clostridium perfringens*)
00047	2	0	2	Firmicutes (g_*Pseudoramibacter_Eubacterium*)
00050	7	0	7	Firmicutes (*Eubacterium dolichum*)
00061	3	0	3	Firmicutes (g_*Pseudoramibacter_Eubacterium*)
00082	2	0	2	Firmicutes (g_*SMB53*)
00108	19	12	7	Firmicutes (g_*Enterococcus*)
00125	4	0	4	Firmicutes (f_Peptostreptococcaceae)
00132	1	0	1	Bacteroidetes (*Bacteroides fragilis*)
00139	3	0	3	Bacteroidetes (*Parabacteroides distasonis*)
00163	1	1	0	Firmicutes (*Staphylococcus epidermidis*)
00166	1	0	1	Firmicutes (f_Peptostreptococcaceae)
00221	1	0	1	Firmicutes (f_Lachnospira)
00321	2	1	1	Firmicutes (g_*Oscillospira*)
00322	1	1	0	Firmicutes (g_*Dorea*)
00432	1	0	1	Firmicutes (g_*Dorea*)
00457	4	4	0	Firmicutes (g_*Clostridium*)
00647	1	0	1	Bacteroidetes (g_Bacteroides)
00650	5	1	4	Firmicutes (f_Lachnospira)
00688	6	0	6	Bacteroidetes (*Bacteroides fragilis*)
00781	1	0	1	Firmicutes (*Eubacterium dolichum*)
00938	1	0	1	Firmicutes (g_*Oscillospira*)
06572	2	2	0	Firmicutes (g_*Enterococcus*)
Total =	116	45	71	

Values represent the number of isolates matched per OTU. A detailed list of cultured isolates is found in Table S13.

Chitin digestion was observed in one of the 14 isolates tested in this experiment (isolate 15–7, Table S7). The isolate belonged to the bacterial genus *Clostridium*, and was further classified as *Clostridium paraputrificum*, a chitinolytic bacterium previously isolated from human feces^27, 28^, by the NCBI non-redundant database (> 97% identity).

## Discussion

The characterization of gut microbiomes in juvenile kogiids expands our understanding of the composition and development of gut symbionts in *K. sima* and *K. breviceps*. Firmicutes and Bacteroidetes dominated the gut microbiomes of juvenile *K. sima* and *K. breviceps*, consistent with previously characterized adult kogiids^22^, as well as other marine and terrestrial mammals^17, 29^. The species-specific differences in gut microbiomes of *K. sima* and *K. breviceps* reported in adult whales^22^ were also evident early in life. Further, significant shifts in the composition and diversity of symbiont communities were observed between life history stages, including a trend towards microbiome convergence with host maturity. Core gut microbiomes consisted of a relatively small number of highly abundant OTUs, with core communities displaying considerable overlap between juvenile and adult kogiids. In addition, two of these symbionts were successfully isolated in pure culture, confirming that dominant members of the gut community can be cultivated and functionally characterized for a more holistic picture of kogiid microbiomes.

Kogiid whales harbored gut microbiomes differentiated by both host phylogeny and life stage. The composition of adult kogiid microbiomes were more similar (within and between species) than juvenile microbiomes, indicating gut communities converge with host maturity. Gut microbiomes also appeared to stabilize with host maturity, as adults possessed the largest core community (i.e. shared symbionts) and no differences were detected in symbiont richness between adult kogiids. Juvenile kogiids shared 50 of the 54 symbiont OTUs in their core community with the adult core microbiome, indicating juvenile kogiids retain most of their core members, and gain additional members with maturity. The overlap of core symbionts also suggests that core members are established early in life, likely through the vertical transmission of symbionts during birth. Transmission of symbionts during passage through the birth canal is documented in humans and is a primary and significant reservoir for the natal microbiome^30–33^. Additionally, vertical transmission of core symbionts has been documented in southern elephant seal mother–pup pairs^34^. Retention of these core members may indicate that they perform functions critical for host health and homeostasis in the kogiid gut.

Despite the potential for vertical transmission of core gut symbionts, overall gut microbiomes in two cow-calf pairs of *K. breviceps* were no more similar to each other than unrelated juvenile and adult *K. breviceps*, suggesting that calf gut microbiomes may diverge quickly after birth. A similar trend occurs in humans, wherein neonates are exposed to sources of microorganisms immediately after birth, thus stimulating the development of the neonatal microbiome^35^. Sampling the mothers’ vaginal microbiomes and the gut microbiomes of calves of varying ages could clarify how quickly the calf microbiome develops. While such sampling is not feasible with wild cetaceans, the rates of calf microbiome development could be investigated in cetacean species under human care, such as bottlenose dolphins, *Tursiops truncatus*. Interestingly, the unrelated mother gut microbiomes were highly similar to one another in comparison to other *K. breviceps* adults. Kogiid mothers could experience shifts in microbiome composition and diversity throughout gestation and the postpartum period similar to those observed in humans^35–37^ and other mammals^38^. In total, these observations suggest a postpartum disturbance of the gut microbiome in kogiid whales, and the rapid development of the calf gut microbiome after birth.

Accordingly, juvenile and adult gut microbiomes are differentiated by both the relative abundance of shared taxa and by symbiont membership. Juvenile *K. sima* and juvenile *K. breviceps* shared taxa unique to this early life stage, while adult *K. sima* and adult *K. breviceps* shared taxa unique to this mature life stage. In addition to age-related differences in microbiome composition, clear shifts in symbiont diversity were observed between juvenile and adult kogiid whales. Some trends were consistent between the host species, such as lower gut microbiome diversity and evenness in juvenile *K. sima* and *K. breviceps* compared to adults. While juvenile and adult microbiomes were differentiated by community evenness, dominance, and diversity, these distinctions were not present between species, perhaps indicating shifts in microbiome diversity are processes unique to host development. Significantly lower symbiont richness in neonates and infants is well established in humans^30, 39^, and was documented in adult-pup comparisons of southern elephant seals^34^. Neonatal and infant microbiomes likely begin to undergo shifts in diversity soon after birth coinciding with ontogenetic and environmental changes. Other trends differed between hosts and illustrated species-specific methods of community assembly with host maturity. Specifically, symbiont richness increased with age in *K. sima* (i.e. ‘accumulation’) but decreased with age in *K. breviceps* (i.e. ‘winnowing’). While host life stage is a determining factor of gut microbiome diversity, the processes underlying shifts in diversity may be unique to host species. Additional sampling and replication is needed in future studies of these elusive animals to increase the power of statistical inference and document the consistency of this phenomenon.

Host microbiomes are relatively stable over time, but internal life events, such as aging and development, are known to cause shifts in the existing symbiotic communities^15^. These ontogenetic shifts in microbiome composition and diversity are often linked to dietary changes (i.e. transitioning from a milk-based diet to more complex, solid foods), and are well documented in humans^40^. To date and to our knowledge, no comprehensive examinations of age-related effects on deep-diving cetacean microbiomes exist. This study illustrates the influence life history stage exerts over microbiome composition and diversity in two closely related deep-diving cetacean species. Gut microbiomes are highly species-specific with strong taxonomic or functional ties to host phylogeny, but the kogiid microbiome is not static and does appear to respond to life history events, such as change in diet. In other mammals, the development of a healthy microbiome is linked to the development of a healthy host immune system and overall host homeostasis^2–5^. Additional research is needed to resolve the functional consequences of the observed structural shifts in whale microbiomes and will require more in-depth knowledge of symbiont physiology.

Symbiont isolation in pure culture allows for the direct metabolic study and experimentation to elucidate functionality; however, many gut bacteria elude cultivation using traditional media and conditions. By utilizing a recent protocol specific to gut bacteria^26^, we show that dominant, core symbionts can be cultured under anaerobic conditions with enriched media. The majority of isolates belonged to the bacterial phylum Firmicutes, a lineage often characterized as spore-forming, possibly aiding in cultivation success. Certain strains within this lineage are known to form endospores and it is hypothesized the ability to form spores in the mammalian gut aids in host-to-host transmission by protection from the aerobic environment^26, 39^. Notably, culture work from fresh fecal samples produced similar taxa as archived (frozen) samples, including the aforementioned spore-forming lineages. Thus, these isolates may dominate the cultured representatives of the kogiid gut microbiome due to both their high abundance in the community and their likely ability to form spores.

Chitin degradation is a potentially important symbiont function in the kogiid gut, as both *K. sima* and *K. breviceps* primarily eat cephalopods with chitinous beaks and pens^18^. Although mammals do possess chitin degrading enzymes, it is traditionally thought they cannot utilize chitin as a carbon or nitrogen source^41, 42^. In contrast, chitin utilization as a nutrient source by aquatic and soil microbes is well documented, and *Clostridium* and *Bacteroides*, two genera isolated from kogiid feces, are known to possess chitinases^41^. Our methods produced visible chitin digestion in one isolate, classified as *C. paraputrificum*. Chitin may not be the preferred nutrient source for these isolates, or kogiids may regurgitate the beaks after accumulation in the stomach^18^, which may explain the lack of chitin utilization in our other isolates. Kogiid gut symbionts, such as *C. paraputrificum*, may aid in chitin digestion, but further investigations of symbiont chitinases and the potential to utilize this source are needed to make conclusions of their function. Future assays could include investigations into the utilization of milk oligosaccharides in the juvenile kogiid gut microbiome. Evidence in humans suggests infants harbor specific symbionts that digest components of the mother’s milk that would otherwise be indigestible by the infant^43–45^, and a similar trend may exist in kogiid whales. Further analysis of the metabolic potential of cultured dominant symbionts could elucidate their functional role in the kogiid gut.

One of the dominant kogiid symbionts isolated herein matched to *C. perfringens*, a bacterium that has previously been detected in marine mammals, including pure culture isolation from bottlenose dolphin fecal and enteric samples^24^, and detected in Australian fur seal fecal samples via FISH analysis^24, 46^. Cultured isolates may be used to determine the primary pathogen or cause of mortality in cetaceans^47^, and *C. perfringens* has been identified as the primary cause of infection in a number of bottlenose dolphins, both free-range and captive^48^. However, it is worth noting that primary pathogens may exist in both healthy and diseased animals and could potentially exist as part of the normal microflora^24, 49^. The function of common intestinal *Clostridium* spp., including *C. perfringens*, within the cetacean gut has not been explored, and its role as either a member of the normal flora or a pathogen in the kogiid gut is not known. The isolation of kogiid-specific strains of *C. perfringens* and other gut taxa may aid in advancing future, hypothesis-driven studies of functionality.

Kogiid whale morbidity and mortality has been linked to various disease states and anthropogenic factors (e.g. parasitism and plastic ingestion, respectively)^18^, but the specific cause of many strandings is uncertain. The relationship between microbiome dysbiosis and overall health has been linked in humans^50^ and could extend to marine mammals. Combining culture-independent and culture-based methods may clarify kogiid health states through whole microbiome characterizations and the identification of key community members. The characterization of the kogiid gut microbiome and the cultivation of dominant symbionts within this study contributes to the relatively sparse amount of data on kogiid microbiomes and may help elucidate the role of the gut microbiome in the health and disease of these commonly stranded cetaceans.

## Methods

### Sample collection

To characterize the kogiid gut microbiome, fecal samples were recovered during necropsy of stranded *K. breviceps* (n = 18) and *K. sima* (n = 9) from North Carolina and Virginia between 2008 and 2018 (Table S8), and subsequently stored at − 80 °C. Differences in sample number between species is a reflection of a greater number of *K. breviceps* that strand than *K. sima* off the coast of North Carolina^51^. Sampled individuals were from two life history stage classifications, juvenile (*K. breviceps* n = 7, *K. sima* n = 5) and adult (i.e. sexually mature) (*K. breviceps* n = 9, *K. sima* n = 4), and from both sexes. All sampled individuals stranded alone except for two cow-calf pairs (K1–K14, K4–K17). One of the adults was pregnant (K1), and the second (K4) had milk present in her mammary tissues. No individuals exhibited direct human-induced mortality, and all were stranded in fresh to moderate carcass condition (Table S8).

### DNA extraction and sequence processing

DNA extracts were prepared from 200 mg of fecal material with the DNeasy PowerSoil Kit (Qiagen). Microbial communities of juvenile samples (n = 12) were characterized by 16S rRNA gene sequencing (V4 region) on an Illumina MiSeq platform at Molecular Research LP (Shallowater, TX), as described previously for adult samples^22^. Briefly, DNA extracts were used as templates for PCR amplifications with the universal bacterial/archaeal forward primer 515F and reverse primer 806R^52^, pooled in equimolar concentrations, purified and sequenced on an Illumina MiSeq. Combined raw datasets from adult and juvenile samples were processed simultaneous in the mothur software package (version 1.39.5)^53^ as detailed previously^22^. Briefly, sequences were quality-filtered, aligned to the SILVA database, and non-target, chimeric, and singleton sequences were removed. High-quality sequences were grouped to form operational taxonomic units (OTUs) at 97% identity using the OptiClust algorithm in mothur^54^. Consensus sequences were obtained for each OTU and classified with the Greengenes database (May 2013 release, mothur-formatted)^55^. To account for variation in sequence depth, sequences from each fecal sample were subsampled to the lowest read count (29,656 sequences). Sequence data were deposited as FASTQ files in the Sequence Read Archive of the National Center for Biotechnology Information (SRA NCBI) under accession number SUB6765511.

### DNA sequence-based characterization

To compare microbiome similarity between kogiid hosts, beta-diversity calculations were performed based on OTU-dependent metrics (Bray–Curtis similarity) in PRIMER (version 6.1.11, PRIMER-e Ltd.), and OTU-independent metrics (UniFrac distance) in mothur. Beta-diversity metrics were also analyzed based on symbiont relative abundance (OTU relative abundance Bray–Curtis, weighted UniFrac) and symbiont membership (OTU presence-absence Bray–Curtis, unweighted UniFrac) within the microbiome. Significant differences in microbiome similarity across the factors host species (*K. breviceps* vs. *K. sima*), life history stage (juvenile vs. adult) and an interaction term (host species × life history stage) were determined by permutational multivariate analyses of variance (PERMANOVA). Permutation multivariate analyses of dispersion (PERMDISP) were performed to compare dispersion between factors. Two-way analyses of similarity (ANOSIM) were performed in PRIMER to detect differences between sex and carcass condition.

Alpha-diversity calculations for richness (observed richness, *S*), evenness (Simpson, *E*_*1*/*D*_), diversity (inverse Simpson, *1*/*D*), and dominance (Berger–Parker, *d*) were performed in mothur for each sample. Alpha-diversity metrics provided data for the observed number of unique symbionts (OTUs) present (*S*), the similarity in abundance of the unique OTUs (*E*_*1*/*D*_), and the proportional abundance of the most dominant OTUs (*d*). Diversity (*1*/*D*) calculations took into account the number of OTUs present as well as the abundance of each OTU. Significant differences across host species, life history stage and an interaction term were determined for each metric with analyses of variance (ANOVA) in JMP Pro (version 14.0.0, SAS Institute). Significant interaction terms were followed with pairwise post hoc tests among levels of each factor using Tukey’s honest significant difference (HSD) test.

A one-way similarity percentage species contributions (SIMPER) analysis was performed in PRIMER to identify the percent contribution of specific OTUs to the community and the overall similarity or dissimilarity between hosts. MetaStats and LefSe were also conducted in mothur to identify significantly differentially abundant OTUs between host life stages, with OTUs considered differentially abundant if significant (*p* < 0.05) using both statistical methods. Significant differences in relative abundance of phyla were determined by Student’s *t* tests performed in JMP (version 12.0) assuming equal variance unless the test for equal variance failed (*F* test 2-sided, *p* < 0.05) and corrected for multiple comparisons (Benjamini–Yekutieli false-discovery rate control^56^ and an experiment-wise error rate of alpha = 0.05). Core gut communities were identified herein as OTUs present in all sampled host individuals. Core symbiont OTUs were determined for each host species (*K. breviceps* and *K. sima*) and life history stage (adult and juvenile).

### Technical replicates

To assess technical variation across sequencing runs, five samples (K14, 17, 18, 21, and 22) were separately extracted and sequenced, then re-analyzed with the full data set (in place of the original sequencing runs for these five samples). Sequencing results were consistent across runs; for example, all statistical trends in microbiome similarity (beta-diversity) were consistent between data sets (Tables S9–S12). Further, no significant differences in composition (Bray–Curtis similarity) were detected between replicate runs of the same samples when analyzed in isolation (PERMANOVA, *p* = 0.697). Accordingly, replicate data indicate that our findings are robust to technical artefacts and were excluded from final analyses present herein.

### Culture-based characterization

Cultivation work was conducted on the same sample set as above for DNA-based characterization, except for one sample (K12) that lacked sufficient material and two additional, fresh samples (K26, K27) from stranding events that occurred during the tenure of this study and were examined prior to sample storage at − 80 °C (Table S8). Fecal material was homogenized separately and diluted in phosphate-buffered saline (0.1 g of fecal material in 1 mL of solution). Serial tenfold dilutions were performed to 10^–4^ and 100 µl of each dilution was plated on YCFA agar supplemented with 2 g/L of glucose as previously described for isolating anaerobic gut symbionts^26, 57^. All media and materials were kept in anaerobic conditions at least 24 h before use. Plates were incubated in anaerobic conditions using the BD GasPak EZ anaerobe container system at 37 °C.

After 72 h, isolated colonies were streaked for isolation, and pure cultures were preserved in 15% glycerol and stored at − 80 °C. DNA was extracted from pure cultures with the One-4-All Genomic DNA Mini-Preps Kit (Bio Basic). The 16S rRNA gene was amplified using OneTaq Quick-Load 2X MM w/Std Buffer (New England BioLabs) and the universal bacterial/archaeal forward and reverse primers Eco8F (5′-AGAGTTTGATCATGGCTCAG-3′)^58^ and 1509R (5′-GGTTACCTTGTTACGACTT-3′)^59^. Thermocycler conditions were as follows: an initial denaturation step at 95 °C for 39 s; 30 cycles of 95 °C for 15 s, 50 °C for 15 s, and 68 °C for 1.5 min; and a final elongation step at 68 °C for 5 min. PCR products were purified using the EZ Spin Column PCR Products Purification Kit (Bio Basic) and sent to MCLAB (San Francisco, CA) for Sanger sequencing. In addition to the universal forward and reverse primers, the internal primer 515F (5′-GTGCCAGCMGCCGCGGTA-3′)^52^ was used during Sanger sequencing for sequence quality. Forward and reverse reads were assembled in Geneious, and a custom 16S rRNA sequence database was created in Geneious from the OTUs recovered during Illumina sequence processing. Isolate consensus sequences were compared to the custom database to match cultured representatives to OTUs in our culture-independent sequence dataset. Consensus sequences were also analyzed using BLAST in NCBI for further taxonomic classification and sample source information. Final sequences were archived in GenBank under accession numbers MN567482 to MN567597.

Fecal samples from the two kogiids that stranded during the tenure of the study (K26 and K27) were plated on YCFA and Brain Heart Infusion (BD BBL) agar within 24 h of examination and necropsy, but were omitted from DNA sequence-based characterization (Table S8). Further sample processing followed the culture-based methods outlined above.

Following a previously described method^60^, chitin assays were performed to test the ability of isolates to utilize chitin as a nutrient source. A total of 14 isolates were each grown in 5 mL of YCFA for 48–72 h. Isolates were spotted on modified YCFA plates covered with a 2% chitin slurry solution. YCFA agar was modified by reducing glucose and yeast extract components to one-tenth of the original concentration. Plates were incubated anaerobically at 37 °C for seven to ten days. Chitin utilization was visible by a clearing of the 2% chitin slurry solution. *Serratia marcescens*, a facultative anaerobe known to digest chitin^61, 62^, was used as the positive control for chitin utilization.

### Ethics statement

As described in a previous publication^22^, all research activities were carried out under a NOAA Stranding Agreement to UNCW and research protocols were approved by UNCW’s Institutional Animal Care and Use Committee (protocols A0809-019, A1112-013, A1415-015 and A1718-011). There is considerable uncertainty surrounding the status of *Kogia sima* and *K*. *breviceps*, with both species categorized as “Data Deficient” on the IUCN Red List of Threatened Species (https://www.iucnredlist.org). This study relied solely upon postmortem sampling of stranded kogiid whales from North Carolina and Virginia, responded to under authorization of the US Marine Mammal Protection Act. Animals were either found freshly dead (*n* = 7) died during initial response (*n* = 6) or underwent humane euthanasia (n = 12) for reasons unrelated to this study following consultation with the National Marine Fisheries Service and under the supervision of a licensed veterinarian in accordance with the American Veterinary Medical Association Guidelines for the Euthanasia of Animals (2013 Edition).

## Supplementary information

Supplementary Information.

## References

[1] Bordenstein SR, Theis KR (2015). Host biology in light of the microbiome: ten principles of holobionts and hologenomes. PLOS Biol..

[2] Cho I, Blaser MJ (2012). The human microbiome: at the interface of health and disease. Nat. Rev. Genet..

[3] Huttenhower C (2012). Structure, function and diversity of the healthy human microbiome. Nature.

[4] Barko PC, McMichael MA, Swanson KS, Williams DA (2018). The gastrointestinal microbiome: a review. J. Vet. Intern. Med..

[5] Guarner F, Malagelada J-R (2003). Gut flora in health and disease. Lancet.

[6] Shafquat A, Joice R, Simmons SL, Huttenhower C (2014). Functional and phylogenetic assembly of microbial communities in the human microbiome. Trends Microbiol..

[7] Qin J (2010). A human gut microbial gene catalogue established by metagenomic sequencing. Nature.

[8] Yildirim S (2010). Characterization of the fecal microbiome from non-human wild primates reveals species specific microbial communities. PLoS ONE.

[9] Nelson TM, Apprill A, Mann J, Rogers TL, Brown MV (2015). The marine mammal microbiome: current knowledge and future directions. Microbiol. Aust..

[10] Bik EM (2016). Marine mammals harbor unique microbiotas shaped by and yet distinct from the sea. Nat. Commun..

[11] Ley RE (2008). Evolution of mammals and their gut microbes. Science.

[12] Shade A, Handelsman J (2012). Beyond the Venn diagram: the hunt for a core microbiome. Environ. Microbiol..

[13] Moura JF (2016). Stranding events of *Kogia* whales along the Brazilian coast. PLoS ONE.

[14] Avila IC, Kaschner K, Dormann CF (2018). Current global risks to marine mammals: taking stock of the threats. Biol. Conserv..

[15] Apprill A (2017). Marine animal microbiomes: toward understanding host–microbiome interactions in a changing ocean. Front. Mar. Sci..

[16] Sanders JG (2015). Baleen whales host a unique gut microbiome with similarities to both carnivores and herbivores. Nat. Commun..

[17] Nelson TM, Rogers TL, Brown MV (2013). The gut bacterial community of mammals from marine and terrestrial habitats. PLoS ONE.

[18] Staudinger MD, McAlarney RJ, McLellan WA, Ann Pabst D (2014). Foraging ecology and niche overlap in pygmy (*Kogia breviceps*) and dwarf (*Kogia sima*) sperm whales from waters of the U.S. mid-Atlantic coast. Mar. Mammal Sci..

[19] McAlpine D, Perrin W, Würsig B, Thewissen J (2009). Pygmy and dwarf sperm whales: *Kogia breviceps* and *K. sima*. Encyclopedia of Marine Mammals.

[20] Manire CA, Rhinehart HL, Barros NB, Byrd L, Cunningham-Smith P (2004). An approach to the rehabilitation of *Kogia* spp. Aquat. Mamm..

[21] Beatson E (2007). The diet of pygmy sperm whales, *Kogia breviceps*, stranded in New Zealand: implications for conservation. Rev. Fish Biol. Fish..

[22] Erwin PM (2017). High diversity and unique composition of gut microbiomes in pygmy (*Kogia breviceps*) and dwarf (*K. sima*) sperm whales. Sci. Rep..

[23] Adrangi S, Faramarzi MA (2013). From bacteria to human: a journey into the world of chitinases. Biotechnol. Adv..

[24] Lagier J-C (2012). Microbial culturomics: paradigm shift in the human gut microbiome study. Clin. Microbiol. Infect..

[25] Morris PJ (2011). Isolation of culturable microorganisms from free-ranging bottlenose dolphins (*Tursiops truncatus*) from the southeastern United States. Vet. Microbiol..

[26] Buck JD, Wells RS, Rhinehart HL, Hansen LJ (2006). Aerobic microorganisms associated with free-ranging bottlenose dolphins in coastal Gulf of Mexico and Atlantic Ocean waters. J. Wildl. Dis..

[27] Browne HP (2016). Culturing of ‘unculturable’ human microbiota reveals novel taxa and extensive sporulation. Nature.

[28] Simůnek J, Kopecný J, Hodrová B, Bartonová H (2002). Identification and characterization of *Clostridium paraputrificum*, a chitinolytic bacterium of human digestive tract. Folia Microbiol. (Praha).

[29] Evvyernie D (2000). Identification and characterization of *Clostridium paraputrificum* M-21, a chitinolytic, mesophilic and hydrogen-producing bacterium. J. Biosci. Bioeng..

[30] Tap J (2009). Towards the human intestinal microbiota phylogenetic core. Environ. Microbiol..

[31] Koenig JE (2011). Succession of microbial consortia in the developing infant gut microbiome. Proc. Natl. Acad. Sci..

[32] Greenhalgh K, Meyer KM, Aagaard KM, Wilmes P (2016). The human gut microbiome in health: establishment and resilience of microbiota over a lifetime. Environ. Microbiol..

[33] Ferretti P (2018). Mother-to-infant microbial transmission from different body sites shapes the developing infant gut microbiome. Cell Host Microbe.

[34] Dominguez-Bello MG (2010). Delivery mode shapes the acquisition and structure of the initial microbiota across multiple body habitats in newborns. Proc. Natl. Acad. Sci..

[35] Nelson TM, Rogers TL, Carlini AR, Brown MV (2013). Diet and phylogeny shape the gut microbiota of Antarctic seals: a comparison of wild and captive animals. Environ. Microbiol..

[36] Mutic AD (2017). The postpartum maternal and newborn microbiomes. MCN Am. J. Matern. Nurs..

[37] Smid M (2018). Maternal gut microbiome biodiversity in pregnancy. Am. J. Perinatol..

[38] Aagaard K (2012). A metagenomic approach to characterization of the vaginal microbiome signature in pregnancy. PLoS ONE.

[39] Antwis RE, Edwards KL, Unwin B, Walker SL, Shultz S (2019). Rare gut microbiota associated with breeding success, hormone metabolites and ovarian cycle phase in the critically endangered eastern black rhino. Microbiome.

[40] Rodríguez JM (2015). The composition of the gut microbiota throughout life, with an emphasis on early life. Microb. Ecol. Health Dis..

[41] Odamaki T (2016). Age-related changes in gut microbiota composition from newborn to centenarian: a cross-sectional study. BMC Microbiol..

[42] Boot RG (2001). Identification of a novel acidic mammalian chitinase distinct from chitotriosidase. J. Biol. Chem..

[43] German, J. B., Freeman, S. L., Lebrilla, C. B. & Mills, D. A. Human milk oligosaccharides: evolution, structures and bioselectivity as substrates for intestinal bacteria. In: *Nestlé Nutrition Workshop Series: Pediatric Program* Vol. 62 (eds. Bier, D. M., German, J. B. & Lönnerdal, B.) 205–222 (KARGER, 2008). 10.1159/000146322 PMC2861563 18626202

[44] Katayama T (2016). Host-derived glycans serve as selected nutrients for the gut microbe: human milk oligosaccharides and bifidobacteria. Biosci. Biotechnol. Biochem..

[45] Underwood MA, German JB, Lebrilla CB, Mills DA (2015). *Bifidobacterium longum* subspecies *infantis*: champion colonizer of the infant gut. Pediatr. Res..

[46] Smith SC, Chalker A, Dewar ML, Arnould JPY (2013). Age-related differences revealed in Australian fur seal *Arctocephalus pusillus doriferus* gut microbiota. FEMS Microbiol. Ecol..

[47] Venn-Watson S, Smith C, Jensen E (2008). Primary bacterial pathogens in bottlenose dolphins *Tursiops truncatus*: needles in haystacks of commensal and environmental microbes. Dis. Aquat. Organ..

[48] Danil K (2014). *Clostridium perfringens* septicemia in a long-beaked common dolphin *Delphinus capensis*: an etiology of gas bubble accumulation in cetaceans. Dis. Aquat. Organ..

[49] Johnson WR (2009). Novel diversity of bacterial communities associated with bottlenose dolphin upper respiratory tracts. Environ. Microbiol. Rep..

[50] Shreiner AB, Kao JY, Young VB (2015). The gut microbiome in health and in disease. Curr. Opin. Gastroenterol..

[51] Byrd BL (2014). Strandings as indicators of marine mammal biodiversity and human interactions off the coast of North Carolina. Fish. Bull..

[52] Caporaso JG (2011). Global patterns of 16S rRNA diversity at a depth of millions of sequences per sample. Proc. Natl. Acad. Sci..

[53] Schloss PD (2009). Introducing mothur: open-source, platform-independent, community-supported software for describing and comparing microbial communities. Appl. Environ. Microbiol..

[54] Westcott SL, Schloss PD (2016). OptiClust: improved method for assigning amplicon-based sequence data to operational taxonomic units. bioRxiv.

[55] DeSantis TZ (2006). Greengenes, a chimera-checked 16S rRNA gene database and workbench compatible with ARB. Appl. Environ. Microbiol..

[56] Benjamini Y, Yekutieli D (2001). The control of false discovery rate in multiple testing under dependency. Ann. Stat..

[57] Duncan SH (2002). Growth requirements and fermentation products of *Fusobacterium prausnitzii*, and a proposal to reclassify it as *Faecalibacterium prausnitzii* gen. nov., comb. nov.. Int. J. Syst. Evol. Microbiol..

[58] Reysenbach AL, Wickham GS, Pace NR (1994). Phylogenetic analysis of the hyperthermophilic pink filament community in Octopus Spring Yellowstone National Park. Appl. Environ. Microbiol..

[59] Martinez-Murcia AJ, Acinas SG, Rodriguez-Valera F (1995). Evaluation of prokaryotic diversity by restrictase digestion of 16s rDNA directly amplified from hypersaline environments. FEMS Microbiol. Ecol..

[60] McBride MJ, Braun TF, Brust JL (2003). *Flavobacterium johnsoniae* GldH is a lipoprotein that Is required for gliding motility and chitin utilization. J. Bacteriol..

[61] Bhattacharya D, Nagpure A, Gupta R (2007). Bacterial chitinases: properties and potential. Crit. Rev. Biotechnol..

[62] Hejazi A, Falkiner F (1997). Serratia marcescens. J. Med. Microbiol..

