# Supplementary material for: Host phylogeny and life history stage shape the gut microbiome in dwarf (Kogia sima) and pygmy (Kogia breviceps) sperm whales
Source: Sci Rep. 2020 Sep 16;10:15162. doi: 10.1038/s41598-020-72032-4 (PMC7495435; doi:10.1038/s41598-020-72032-4)
Supplement: Supplementary file 1 — Supplementary Information. [file 41598_2020_72032_MOESM1_ESM.pdf]

SUPPLEMENATRY MATERIAL FOR:

Host phylogeny and life history stage shape the gut microbiome in dwarf (*Kogia sima*) and pygmy (*Kogia breviceps*) sperm whales

Elizabeth R. Denison, Ryan G. Rhodes, William A. McLellan, D. Ann Pabst, Patrick M. Erwin*

Department of Biology and Marine Biology, Center for Marine Science, University of North Carolina Wilmington, Wilmington, NC 28409, USA

*Corresponding author: erwinp@uncw.edu, tel. 910-962-2326, fax 910-962-2410

Table S1. Relative abundance of bacterial and archaeal phyla in the gut microbiomes of all 25 *K. sima* and *K. breviceps* samples. Relative abundance reported as a percentage (±SD).

**Phylum**	**Relative Abundance**
Firmicutes	45.57 ± 17.09
Bacteroidetes	28.95 ± 15.62
Actinobacteria	11.22 ± 11.20
Proteobacteria	6.03 ± 5.30
Synergistetes	2.56 ± 1.99
Verrucomicrobia	1.53 ± 1.63
Lentisphaerae	0.50 ± 1.04
Tenericutes	0.47 ± 0.66
Euryarchaeota	0.18 ± 0.41
Cyanobacteria	0.04 ± 0.09
Spirochaetes	0.03 ± 0.09
Fusobacteria	0.02 ± 0.06

Table S2. Comparisons of relative abundance (±SD) of bacterial and archaeal phyla in kogiid gut microbiomes between life history stages (juvenile and adult) within each host species (*K. sima*, and *K. breviceps*). Asterisks (*) indicate phyla exhibiting significant differences in relative abundances between hosts following B-Y corrections. All phyla belong to the domain Bacteria, expect Euryarchaeota from the domain Archaea.

	Kogia sima			***Kogia breviceps***		
**Phylum**	Juvenile	Adult	*P*	Juvenile	Adult	*P*
Firmicutes	44.38 ±28.58	61.42 ±18.7	0.3406	37.74 ±11.63	45.27 ±7.44	0.137
Bacteroidetes	19.29 ±17.85	13.00 ±9.72	0.5496	39.9 ±14.79	32.88 ±8.09	0.244
Actinobacteria	25.37 ±17.12	14.34 ±6.08	0.2639	6.58 ±4.75	5.59 ±3.77	0.648
Proteobacteria	8.51 ±7.38	6.85 ±7.87	0.7539	5.60 ±4.38	4.61 ±3.58	0.628
Synergistetes	0.48 ±0.78	2.16 ±2.17	0.1468	2.69 ±2.15	3.80 ±1.33	0.224
Verrucomicrobia	1.25 ±1.75	0.43 ±0.67	0.4092	0.78 ±0.65	2.75 ±1.78	0.011*
Lentisphaerae	0.10 ±0.15	0.04 ±0.02	0.3833	1.18 ±1.84	0.41 ±0.30	0.310
Euryarchaeota	0.01 ±0.02	0.02 ±0.02	0.9779	0.44 ±0.69	0.16 ±0.24	0.336
Tenericutes	0.01 ±0.01	0.56 ±1.03	0.3648	0.41 ±0.50	0.73 ±0.70	0.327
Spirochaetes	0.01 ±0.01	0.00 ±0.01	0.7403	0.08 ±0.18	0.03 ±0.03	0.526
Cyanobacteria	0.01 ±0.01	0.07 ±0.10	0.3064	0.07 ±0.15	0.01 ±0.01	0.354
Fusobacteria	0.00 ±0.00	0.01 ±0.01	0.4071	0.02 ±0.03	0.05 ±0.10	0.511

Table S3. Core OTUs in the kogiid gut microbiomes. Values represent total number of subsampled sequences. OTUs in bold represent the 50 OTUs present in the core of all 25 individuals. Top 25 OTUs (Y) were also found in the top 25 most abundant OTUs across all 25 samples.

OTU	Juvenile Core	Adult Core	Top 25	Total	Phylum (lowest taxonomy)
**00001**	Y	Y	Y	65963	Bacteroidetes (p__Bacteroidetes)
**00002**	Y	Y	Y	70972	Bacteroidetes (p__Bacteroidetes)
**00003**	Y	Y	Y	40099	Firmicutes (f__Peptostreptococcaceae)
**00004**	Y	Y	Y	16664	Firmicutes (f__Peptostreptococcaceae)
**00005**	Y	Y	Y	22556	Actinobacteria (g__*Adlercreutzia*)
**00006**	Y	Y	Y	16324	Firmicutes (*Clostridium perfringens*)
**00007**	Y	Y	Y	23108	Firmicutes (f__Mogibacteriaceae)
**00008**	Y	Y	Y	19344	Firmicutes (o__Clostridia)
**00009**	Y	Y	Y	13079	Bacteroidetes (p__Bacteroidetes)
**00010**	Y	Y	Y	15453	Synergistetes (f__Synergistaceae)
**00011**	Y	Y	Y	22130	Actinobacteria (g__*Mycobacterium*)
**00012**	Y	Y	Y	18696	Bacteroidetes (o__Bacteroidales)
**00013**	Y	Y	Y	17260	Firmicutes (o__Clostridia)
**00014**	Y	Y	Y	10861	Firmicutes (o__Clostridia)
**00015**	Y	Y	Y	14323	Bacteroidetes (p__Bacteroidetes)
**00016**	Y	Y	Y	8213	Proteobacteria (f__Enterobacteriaceae)
**00017**	Y	Y	Y	7862	Firmicutes (g__*Oscillospira*)
**00018**	Y	Y	Y	9423	Firmicutes (g__*Oscillospira*)
00019	-	Y	Y	7716	Proteobacteria (*Campylobacter fetus*)
**00020**	Y	Y	Y	8998	Verrucomicrobia (f__RFP12)
**00021**	Y	Y	Y	10187	Firmicutes (f__Lachnospira)
**00022**	Y	Y	Y	14209	Proteobacteria (g__*Desulfovibrio*)
**00023**	Y	Y	-	6534	Bacteria (k__Bacteria)
**00024**	Y	Y	-	4155	Actinobacteria (f__Coriobacteriaceae)
**00025**	Y	Y	Y	7544	Actinobacteria (f__Coriobacteriaceae)
**00026**	Y	Y	-	5305	Proteobacteria (p__Proteobacteria)
**00027**	Y	Y	-	6651	Firmicutes (f__EtOH8)
**00028**	Y	Y	-	5910	Firmicutes (f__Mogibacteriaceae)
00029	-	Y	-	4858	Bacteria (k__Bacteria)

OTU	Juvenile Core	Adult Core	Top 25	Total	Phylum (lowest taxonomy)
**00030**	Y	Y	Y	8723	Actinobacteria (f__Coriobacteriaceae)
**00031**	Y	Y	-	4401	Firmicutes (o__Clostridia)
00032	-	Y	-	3415	Firmicutes (*Clostridium perfringens*)
00033	-	Y	-	2619	Synergistetes (g__*Synergistes*)
00035	-	Y	-	2051	Firmicutes (*Blautia producta*)
00036	-	Y	-	4205	Proteobacteria (g__*Citrobacter*)
**00037**	Y	Y	Y	8768	Firmicutes (f__Ruminococcaceae)
**00038**	Y	Y	-	4596	Firmicutes (*Faecalibacterium prasnitzii*)
00039	-	Y	-	4934	Bacteroidetes (p__Bacteroidetes)
**00040**	Y	Y	-	4217	Firmicutes (g__*Anaerovorax*)
**00041**	Y	Y	-	4300	Firmicutes (f__Ruminococcaceae)
00042	-	Y	-	3280	Lentisphaerae (f__Victivallaceae)
00043	-	Y	-	3583	Firmicutes (p__Firmicutes)
00044	-	Y	-	1330	Bacteroidetes (p__Bacteroidetes)
**00045**	Y	Y	-	6442	Firmicutes (f__Mogibacteriaceae)
**00046**	Y	Y	-	4376	Actinobacteria (f__Coriobacteriaceae)
00047	-	Y	-	2896	Firmicutes (g__*Pseudoramibacter_Eubacterium*)
**00048**	Y	Y	-	2125	Firmicutes (f__Ruminococcaceae)
00049	-	Y	-	3371	Firmicutes (o__Clostridia)
00050	-	Y	-	1892	Firmicutes (*Eubacterium dolichum*)
**00051**	Y	Y	-	4480	Bacteroidetes (g__*Odoribacter*)
**00052**	Y	Y	-	2747	Proteobacteria (f__0319-6G20)
00053	-	Y	-	2876	Firmicutes (p__Firmicutes)
00054	-	Y	-	1270	Firmicutes (o__Clostridia)
**00056**	Y	Y	-	5152	Firmicutes (f__Ruminococcaceae)
00057	-	Y	-	2816	Firmicutes (f__Erysipelotrichaceae)
00058	-	Y	-	2403	Actinobacteria (*Atopobium rimae*)
00059	-	Y	-	2626	Firmicutes (o__Clostridia)
00060	-	Y	-	2078	Firmicutes (f__Lachnospira)
**00063**	Y	Y	-	3117	Actinobacteria (f__Coriobacteriaceae)
00064	-	Y	-	1885	Firmicutes (g__*Ruminococcus*)
**00065**	Y	Y	-	1914	Firmicutes (g__*RFN20*)
00066	-	Y	-	2183	Actinobacteria (*Eggerthella lenta*)
00067	-	Y	-	2382	Firmicutes (g__*Syntrophomonas*)
00068	-	Y	-	2166	Firmicutes (o__Clostridia)
00069	-	Y	-	1936	Firmicutes (g__*Oscillospira*)
00070	-	Y	-	2028	Firmicutes (o__Clostridia)
**00071**	Y	Y	-	3665	Firmicutes (*Oscillospira guilliermondii*)

OTU	Juvenile Core	Adult Core	Top 25	Total	Phylum (lowest taxonomy)
00072	-	Y	-	1652	Firmicutes (f__Erysipelotrichaceae)
00074	-	Y	-	1436	Tenericutes (p__Tenericutes)
00075	-	Y	-	1668	Actinobacteria (*Eggerthella lenta*)
00076	-	Y	-	1921	Bacteria (k__Bacteria)
**00077**	Y	Y	-	1717	Firmicutes (g__*Butyrivibrio*)
00079	-	Y	-	1480	Firmicutes (o__Clostridia)
00080	Y	-	-	3142	Firmicutes (*Ruminococcus gnavus)*
00081	-	Y	-	1907	Firmicutes (g__*Syntrophomonas*)
00082	-	Y	-	1630	Firmicutes (g__*SMB53*)
00086	-	Y	-	1546	Firmicutes (f__Christensenellaceae)
00088	-	Y	-	884	Firmicutes (f__Mogibacteriaceae)
00089	Y	-	-	1273	Bacteroidetes (p__Bacteroidetes)
**00090**	Y	Y	-	1115	Actinobacteria (g__*Adlercreutzia*)
00094	-	Y	-	972	Firmicutes (p__Firmicutes)
00097	-	Y	-	648	Bacteria (k__Bacteria)
**00098**	Y	Y	-	919	Firmicutes (g__*Butyrivibrio*)
00099	-	Y	-	779	Firmicutes (g__*Blautia*)
00100	-	Y	-	943	Firmicutes (g__*Butyrivibrio*)
00102	-	Y	-	772	Bacteria (k__Bacteria)
00103	-	Y	-	864	Firmicutes (*Ruminococcus gnavus*)
00104	-	Y	-	827	Firmicutes (g__*Dorea*)
00106	-	Y	-	548	Bacteria (k__Bacteria)
**00110**	Y	Y	-	1025	Firmicutes (g__*Butyrivibrio*)
00113	-	Y	-	657	Firmicutes (*Ruminococcus gnavus*)
**00114**	Y	Y	-	754	Firmicutes (g__*Butyrivibrio*)
00115	-	Y	-	514	Firmicutes (o__Clostridia)
00117	-	Y	-	840	Firmicutes (f__Lachnospira)
**00118**	Y	Y	-	460	Firmicutes (o__Clostridia)
00119	-	Y	-	269	Bacteria (k__Bacteria)
00122	-	Y	-	443	Firmicutes (f__Ruminococcaceae)
00124	-	Y	-	510	Firmicutes (g__*Butyrivibrio*)
00127	-	Y	-	503	Firmicutes (o__Clostridia)
**00128**	Y	Y	-	598	Bacteroidetes (p__Bacteroidetes)
00129	-	Y	-	497	Tenericutes (o__RF39)
00130	-	Y	-	466	Firmicutes (*Ruminococcus gnavus*)
00131	-	Y	-	345	Firmicutes (g__*Oscillospira*)
00132	-	Y	-	338	Bacteroidetes (*Bacteroides fragilis*)
00136	-	Y	-	340	Firmicutes (f__Lachnospira)

OTU	Juvenile Core	Adult Core	Top 25	Total	Phylum (lowest taxonomy)
00137	Y	-	-	1060	Firmicutes (o__Clostridia)
00138	-	Y	-	582	Firmicutes (g__*Ruminococcus*)
00140	-	Y	-	437	Firmicutes (o__Clostridia)
00142	-	Y	-	340	Firmicutes (f__Mogibacteriaceae)
00145	-	Y	-	710	Firmicutes (g__*Ruminococcus*)
00148	-	Y	-	186	Firmicutes (g__*Dorea*)
**00150**	Y	Y	-	613	Firmicutes (g__*Ruminococcus*)
00152	-	Y	-	410	Firmicutes (o__Clostridia)
00157	-	Y	-	245	Firmicutes (f__Mogibacteriaceae)
00158	-	Y	-	477	Firmicutes (f__Ruminococcaceae)
00161	-	Y	-	407	Firmicutes (p__Firmicutes)
00162	-	Y	-	294	Firmicutes (o__Clostridia)
00164	-	Y	-	292	Firmicutes (g__*Ruminococcus*)
00169	-	Y	-	505	Bacteroidetes (g__*Odoribacter*)
00171	-	Y	-	259	Firmicutes (*Blautia producta*)
00173	-	Y	-	230	Proteobacteria (g__*Desulfovibrio*)
00174	-	Y	-	272	Firmicutes (g__*Butyrivibrio*)
00175	-	Y	-	187	Bacteroidetes (f__Rikenellaceae)
00177	-	Y	-	226	Firmicutes (f__Lachnospira)
00179	-	Y	-	296	Firmicutes (g__*Ruminococcus*)
00184	-	Y	-	201	Firmicutes (g__*Oscillospira*)
00186	-	Y	-	223	Firmicutes (g__*Coprococcus*)
00192	-	Y	-	169	Firmicutes (g__*Oscillospira*)
00193	-	Y	-	191	Firmicutes (f__Christensenellaceae)
00196	-	Y	-	98	Firmicutes (f__Christensenellaceae)
00212	-	Y	-	183	Firmicutes (g__*Ruminococcus*)
00224	-	Y	-	139	Bacteria (k__Bacteria)
00225	-	Y	-	167	Firmicutes (g__*Butyrivibrio*)
00226	Y	-	-	114	Bacteroidetes (p__Bacteroidetes)
00229	-	Y	-	148	Proteobacteria (g__*Desulfovibrio*)
00240	-	Y	-	140	Firmicutes (g__*Anaerovorax*)
00249	-	Y	-	106	Firmicutes (g__*Butyrivibrio*)
00268	-	Y	-	108	Firmicutes (g__*Dialister*)
00272	-	Y	-	70	Actinobacteria (p__Actinobacteria)
00285	-	Y	-	61	Firmicutes (g__*Butyrivibrio*)

Table S4. The 25 most common OTUs and their relative abundance in juvenile and adult kogiid whales. Percent contributions to dissimilarity between life history stages (% Contrib.) determined by SIMPER analysis. Values in bold represent differentially abundant symbionts between life history stages (*P*<0.05 for MetaStats and LefSe analyses).

		Kogia sima			***Kogia breviceps***		
**OTU**	**Lowest Taxonomy**	Juvenile	Adult	% Contrib.	Juvenile	Adult	% Contrib.
00001	p__Bacteroidetes	1.24 ±2.01	0.79 ±0.51	0.79	16.71 ±12.34	10.68 ±7.36	10.46
00002	p__Bacteroidetes	8.46 ±9.18	8.41 ±6.41	5.00	7.83 ±6.47	12.07 ±2.20	6.14
00003	f__Peptostreptococcaceae	0.09 ±0.03	0.89 ±0.62	0.49	7.51 ±8.11	8.74 ±6.27	7.18
00004	f__Peptostreptococcaceae	3.86 ±8.23	5.89 ±6.22	4.39	**0.06 ±0.03**	**1.43 ±1.85**	**1.28**
00005	g__*Adlercreutzia*	11.03 ±17.15	3.33 ±1.97	6.96	0.61 ±0.78	0.37 ±0.15	0.51
00006	*Clostridium perfringens*	0.25 ±0.55	8.83 ±11.00	5.42	0.51 ±1.25	1.66 ±2.65	1.68
00007	f__Mogibacteriaceae	0.07 ±0.03	1.56 ±1.83	0.92	3.71 ±2.68	5.04 ±1.46	2.41
00008	o__Clostridia	**0.81 ±1.08**	**6.82 ±2.47**	**3.71**	**0.66 ±0.45**	**3.25 ±3.91**	**2.46**
00009	p__Bacteroidetes	0.71 ±1.02	0.38 ±0.41	0.46	3.76 ±4.42	1.41 ±1.09	2.88
00010	f__Synergistaceae	0.30 ±0.62	0.14 ±0.16	0.21	2.52 ±2.02	3.60 ±1.28	1.90
00011	g__*Mycobacterium*	2.43 ±4.33	1.28 ±1.11	1.67	3.23 ±3.32	3.86 ±3.77	3.50
00012	o__Bacteroidales	0.19 ±0.30	0.08 ±0.06	0.10	4.57 ±3.68	3.31 ±1.99	2.79
00013	o__Clostridia	4.75 ±9.35	3.90 ±3.74	3.88	0.99 ±1.66	1.32 ±1.31	1.34
00014	o__Clostridia	0.13 ±0.15	0.21 ±0.12	0.10	3.01 ±2.53	1.57 ±1.57	2.23
00015	p__Bacteroidetes	3.07 ±6.8	1.49 ±2.67	2.42	1.41 ±1.82	1.91 ±2.26	1.79
00016	f__Enterobacteriaceae	3.91 ±6.88	0.42 ±0.46	2.45	0.06 ±0.06	0.68 ±1.30	0.62
00017	g__*Oscillospira*	0.19 ±0.37	2.36 ±2.91	1.36	**0.47 ±0.38**	**1.43 ±0.62**	**0.96**
00018	g__*Oscillospira*	0.42 ±0.62	0.78 ±0.52	0.41	**0.52 ±0.63**	**2.54 ±1.41**	**1.93**
00019	*Campylobacter fetus*	0.00 ±0.00	0.05 ±0.01	0.03	0.39 ±1.02	2.56 ±3.38	2.35
00020	f__RFP12	0.34 ±0.74	0.33 ±0.50	0.32	0.71 ±0.60	2.48 ±1.72	1.79
00021	f__Lachnospira	0.05 ±0.02	0.27 ±0.07	0.14	2.82 ±3.22	1.48 ±0.62	2.06
00022	g__*Desulfovibrio*	3.65 ±7.00	1.34 ±1.67	2.47	2.88 ±5.02	0.46 ±0.51	2.39
00025	f__Coriobacteriaceae	3.37 ±6.84	1.99 ±1.48	2.56	0.01 ±0.01	0.06 ±0.03	0.05
00030	f__Coriobacteriaceae	4.22 ±8.77	1.91 ±2.74	3.10	0.02 ±0.01	0.05 ±0.03	0.03
00037	f__Ruminococcaceae	5.17 ±9.45	0.06 ±0.06	3.17	0.45 ±0.99	0.04 ±0.06	0.41

Table S5. Pairwise statistical comparison of multivariate dispersion (PERMDISP) based on OTU-dependent (Bray Curtis) and OTU-independent (UniFrac) metrics. Includes OTU relative abundance (Rel. Abund., Weighted) and OTU-independent (Presence-Abs., Unweighted) metrics. Asterisks (*) indicate significant differences.

	**Bray-Curtis Similarity**				**UniFrac Distance**			
	Rel. Abund		Presence-Abs.		Weighted		Unweighted	
**Pairwise Comparison**	*t*	*P*	*t*	*P*	*t*	*P*	*t*	*P*
K. sima vs. *K. breviceps*								
- Both life history stages	2.245	<0.001*	1.446	0.250	4.416	<0.001*	1.117	0.410
- Adults	3.884	0.004*	0.4357	1.000	2.829	0.027*	3.229	0.847
- Juveniles	3.681	0.005*	0.5451	0.731	2.907	0.038*	0.433	0.920
Adults vs. Juveniles								
- Both host species	1.887	<0.001*	12.455	<0.001*	2.395	0.071	12.634	<0.001*
- *K. breviceps*	6.181	<0.001*	10.136	<0.001*	2.127	0.047*	11.283	<0.001*
- *K. sima*	6.045	0.016*	11.036	0.015*	2.651	0.066	15.937	0.009*

Table S6. Average number of observed OTUs within kogiid hosts after subsampling to the lowest read count (±SE).

**Host**	**No. OTUs**
All Juveniles	712 ±44
- *K. sima*	556 ±31
- *K. breviceps*	824 ±24
All Adults	639 ±9
- *K. sima*	645 ±24
- *K. breviceps*	637 ±10
All Individuals	
- *K. sima*	595 ±25
- *K. breviceps*	719 ±26

Table S7. Cultured isolates from fecal samples used in chitin utilization assays. Isolate positive for chitin digestion in bold.

Isolate ID	OTU	Phylum (lowest taxonomy)
11-7	00132	Bacteroidetes (*Bacteroides fragilis*)
7-2	00139	Bacteroidetes (*Parabacteroides distasonis*)
B26-10	00616	Bacteroidetes (g__*Odoribacter*)
B26-9	00647	Bacteroidetes (g__*Bacteroides*)
8-5	00004	Firmicutes (f__Peptostreptococcaceae)
5-1	00004	Firmicutes (f__Peptostreptococcaceae)
B26-4	00047	Firmicutes (g__*Pseudoramibacter Eubacterium*)
8-1	00082	Firmicutes (g__*SMB53*)
12-3	00108	Firmicutes (g__*Enterococcus*)
13-3	00221	Firmicutes (f__Lachnospira)
2-2	00321	Firmicutes (g__*Oscillospira*)
1-5	00432	Firmicutes (g__*Dorea*)
**15-7**	**00457**	**Firmicutes (g__*Clostridium*)**
8-1	00082	Firmicutes (g__*SMB53*)

Table S8. Stranded juvenile and adult *K. breviceps* and *K. sima* used in this study. Carcass condition at stranding (left) and at sample collection (right): 1 = Alive, 2 = Fresh Dead, 3 = Moderate Decomposition. All animals were sampled within 24 hours of stranding, except for one individual sampled 36 hours after death and three carcasses frozen immediately for later sampling. Time of death to sample collection was within 6-36 hours for euthanized animals. Strandings that were found dead were estimated to have died within 24-72 hours of discovery.

Lab ID	Species	Life Stage	Sex	Total Length (cm)	Strand Date	Condition
K15	*K. breviceps*	Juvenile	F	231.5	8-Mar-12	1, 2
K14	*K. breviceps*	Juvenile	F	150	16-Sep-11	1, 2
K16	*K. breviceps*	Juvenile	M	249	18-Nov-14	1, 2
K20	*K. breviceps*	Juvenile	M	225	22-Nov-07	1, 2
K19	*K. breviceps*	Juvenile	M	223.5	24-Apr-10	1, 2
K18	*K. breviceps*	Juvenile	M	201	25-May-10	1, 2
K17	*K. breviceps*	Juvenile	M	149	16-Sep-14	1, 2
K3	*K. breviceps*	Adult	F	296	14-Oct-11	1, 2
K4	*K. breviceps*	Adult	F	295	16-Sep-14	1, 2
K1	*K. breviceps*	Adult	F	286	16-Sep-11	1, 2
K2	*K. breviceps*	Adult	F	252.5	5-Oct-12	1, 2
K27	*K. breviceps*	Adult	F	234.5	11-Dec-18	1, 2
K6	*K. breviceps*	Adult	M	328.5	9-Dec-12	1, 3
K7	*K. breviceps*	Adult	M	307	16-Aug-08	1, 2
K9	*K. breviceps*	Adult	M	293.5	1-Oct-14	1, 2
K26	*K. breviceps*	Adult	M	279	26-Apr-18	1, 2
K8	*K. breviceps*	Adult	M	263.5	15-Dec-09	2, 3
K5	*K. breviceps*	Adult	M	261	3-May-11	1, 2
K25	*K. sima*	Juvenile	M	166	4-Jan-11	1, 2
K24	*K. sima*	Juvenile	M	156	11-Dec-13	1, 2
K23	*K. sima*	Juvenile	M	116.5	16-Oct-15	3, 3
K21	*K. sima*	Juvenile	M	116.1	14-Jan-15	3, 3
K22	*K. sima*	Juvenile	M	114.1	3-Jul-07	2, 2
K12	*K. sima*	Adult	M	236.5	24-Aug-10	2, 3
K10	*K. sima*	Adult	F	233.5	24-Aug-10	2, 3
K11	*K. sima*	Adult	M	226	6-Jul-11	1, 2
K13	*K. sima*	Adult	M	220	25-Aug-10	2, 3

Table S9. Pairwise statistical comparisons of microbiome similarity based on Bray Curtis similarity of relative abundance (Rel. Abund.) and presence-absence (Presence-Abs.) data. Final data (Final) and technical replicates (Replicates) are shown. Asterisks (*) indicate significant differences.

	**Final**				**Replicates**			
	Rel. Abund		Presence-Abs.		Rel. Abund		Presence-Abs.	
**Pairwise Comparison**	*t*	*P*	*t*	*P*	*t*	*P*	*t*	*P*
K. breviceps vs. *K. sima*								
- Both life history stages	2.245	<0.001*	1.532	<0.001*	2.311	<0.001*	1.488	<0.001*
- Adults	1.999	0.001*	1.279	0.002*	1.999	0.002*	1.279	0.001*
- Juveniles	1.654	0.003*	1.409	0.002*	1.709	0.003*	1.312	0.008*
Adults vs. Juveniles								
- Both host species	1.886	<0.001*	1.832	<0.001*	1.877	<0.001*	1.822	<0.001*
- *K. breviceps*	1.730	<0.001*	1.710	<0.001*	1.689	<0.001*	1.630	<0.001*
- *K. sima*	1.295	0.032*	1.380	0.014*	1.271	0.038*	1.350	0.018*

Table S10. Pairwise statistical comparisons of microbiome similarity based on UniFrac distance of relative abundance (Weighted) and presence-absence (Unweighted) data. Final data (Final) and technical replicates (Replicates) are shown. Asterisks (*) indicate significant differences.

	**Final**				**Replicates**			
	Weighted		Unweighted		Weighted		Unweighted	
**Pairwise Comparison**	*t*	*P*	*t*	*P*	*t*	*P*	*t*	*P*
K. breviceps vs. *K. sima*								
- Both life history stages	2.727	<0.001*	1.278	0.002*	2.919	<0.001*	1.277	0.001*
- Adults	2.581	0.002*	1.157	0.001*	2.581	0.002*	1.157	0.001*
- Juveniles	1.723	0.007*	1.178	<0.001*	1.960	0.005*	1.154	0.026*
Adults vs. Juveniles								
- Both host species	1.456	0.035*	1.439	<0.001*	1.395	0.059	1.489	<0.001*
- *K. breviceps*	1.202	0.151	1.352	0.001*	1.432	0.059	1.343	<0.001*
- *K. sima*	1.053	0.341	1.175	0.007*	0.941	0.547	1.197	0.024*

Table S11. Pairwise statistical comparisons of multivariate dispersion (PERMDISP) based on Bray Curtis similarity of relative abundance (Rel. Abund.) and presence-absence (Presence-Abs.) data. Final data (Final) and technical replicates (Replicates) are shown. Asterisks (*) indicate significant differences.

	**Final**				**Replicates**			
	Rel. Abund		Presence-Abs.		Rel. Abund		Presence-Abs.	
**Pairwise Comparison**	*t*	*P*	*t*	*P*	*t*	*P*	*t*	*P*
K. breviceps vs. *K. sima*								
- Both life history stages	2.245	<0.001*	1.446	0.250	4.201	0.001*	1.817	0.156
- Adults	3.884	0.004*	0.4357	1.000	3.884	0.003*	0.436	1.000
- Juveniles	3.681	0.005*	0.5451	0.731	3.676	0.018*	1.325	0.306
Adults vs. Juveniles								
- Both host species	1.887	<0.001*	12.455	<0.001*	5.089	0.004*	1.279	0.002*
- *K. breviceps*	6.181	<0.001*	10.136	<0.001*	4.365	0.005*	6.621	<0.001*
- *K. sima*	6.045	0.016*	11.036	0.015*	7.467	0.024*	13.74	0.016*

Table S12. Pairwise statistical comparisons of multivariate dispersion (PERMDISP) based on UniFrac distance of relative abundance (Weighted) and presence-absence (Unweighted) data. Final data (Final) and technical replicates (Replicates) are shown. Asterisks (*) indicate significant differences.

	**Final**				**Replicates**			
	Weighted		Unweighted		Weighted		Unweighted	
**Pairwise Comparison**	*t*	*P*	*t*	*P*	*t*	*P*	*t*	*P*
K. breviceps vs. *K. sima*								
- Both life history stages	4.416	<0.001*	1.117	0.410	4.409	<0.001*	1.607	0.236
- Adults	2.829	0.027*	3.229	0.847	1.827	0.102	3.229	0.846
- Juveniles	2.907	0.038*	0.4331	0.920	3.329	0.017*	1.4465	0.401
Adults vs. Juveniles								
- Both host species	2.395	0.071	12.634	<0.001*	2.939	0.025*	10.065	<0.001*
- *K. breviceps*	2.127	0.047*	11.283	<0.001*	2.820	0.028*	7.532	<0.001*
- *K. sima*	2.651	0.066	15.937	0.009*	3.489	0.025*	26.73	0.007*

Table S13. All cultured isolates from *K. sima* and *K. breviceps* fecal samples, showing host sources, isolate labels, OTU matches, match percentages and taxonomy.

Host	Isolate	OTU Match	% Pairwise Identity	Phylum (Lowest Taxonomy)
Juvenile	14-1	Otu00006	100	Firmicutes (*Clostridium perfringens*)
	14-2	Otu00032	99.6	Firmicutes (*Clostridium perfringens*)
	14-3	Otu00006	99.6	Firmicutes (*Clostridium perfringens*)
	14-4	Otu00006	100	Firmicutes (*Clostridium perfringens*)
	14-5	Otu00006	99.6	Firmicutes (*Clostridium perfringens*)
	15-1	Otu00006	100	Firmicutes (*Clostridium perfringens*)
	15-2	Otu00322	99.2	Firmicutes (g__Dorea)
	15-3	Otu00321	98.8	Firmicutes (g__Oscillospira)
	15-7	Otu00457	99.6	Firmicutes (g__Clostridium)
	15-8	Otu00006	100	Firmicutes (*Clostridium perfringens*)
	16-1	Otu00004	99.2	Firmicutes (f__Peptostreptococcaceae)
	16-2	Otu00004	100	Firmicutes (f__Peptostreptococcaceae)
	16-2A	Otu00004	100	Firmicutes (f__Peptostreptococcaceae)
	16-3	Otu00006	100	Firmicutes (*Clostridium perfringens*)
	16-3A	Otu00163	99.6	Firmicutes (Staphylococcus epidermidis)
	16-4	Otu00004	100	Firmicutes (f__Peptostreptococcaceae)
	16-5	Otu00006	100	Firmicutes (*Clostridium perfringens*)
	17-1	Otu00004	99.6	Firmicutes (f__Peptostreptococcaceae)
	18-1	Otu00108	100	Firmicutes (g__Enterococcus)
	18-2 F	Otu00108	100	Firmicutes (g__Enterococcus)
	18-2 R	Otu06572	100	Firmicutes (g__Enterococcus)
	18-4	Otu00108	100	Firmicutes (g__Enterococcus)
	18-5 F	Otu00108	100	Firmicutes (g__Enterococcus)
	18-5 R	Otu06572	100	Firmicutes (g__Enterococcus)
	18-7	Otu00108	99.6	Firmicutes (g__Enterococcus)
	20-1	Otu00006	100	Firmicutes (*Clostridium perfringens*)
	20-2	Otu00006	100	Firmicutes (*Clostridium perfringens*)
	20-3	Otu00650	99.2	Firmicutes (f__Lachnospira)
	22-1	Otu00457	99.2	Firmicutes (g__Clostridium)
	22-3	Otu00457	99.2	Firmicutes (g__Clostridium)
	22-4	Otu00457	99.6	Firmicutes (g__Clostridium)
	23-1	Otu00004	100	Firmicutes (f__Peptostreptococcaceae)
	23-2	Otu00004	98.8	Firmicutes (f__Peptostreptococcaceae)
	24-1	Otu00108	100	Firmicutes (g__Enterococcus)

	24-1A	Otu00108	99.6	Firmicutes (g__Enterococcus)
	24-2	Otu00108	99.2	Firmicutes (g__Enterococcus)
	24-2A	Otu00108	99.2	Firmicutes (g__Enterococcus)
	24-3	Otu00108	100	Firmicutes (g__Enterococcus)
	24-4	Otu00108	100	Firmicutes (g__Enterococcus)
	24-5	Otu00108	99.6	Firmicutes (g__Enterococcus)
	25-1	Otu00004	100	Firmicutes (f__Peptostreptococcaceae)
	25-2	Otu00004	100	Firmicutes (f__Peptostreptococcaceae)
	25-3	Otu00004	100	Firmicutes (f__Peptostreptococcaceae)
	25-4	Otu00004	99.6	Firmicutes (f__Peptostreptococcaceae)
Adult	1-1	OTU00061	100	Firmicutes (g__Pseudoramibacter_Eubacterium)
	1-3	OTU00650	99.2	Firmicutes (f__Lachnospira)
	1-4	OTU00061	99.6	Firmicutes (g__Pseudoramibacter_Eubacterium)
	1-5	OTU00432	100	Firmicutes (g__Dorea)
	1-6	OTU00650	99.2	Firmicutes (f__Lachnospira)
	1-8	OTU00650	99.2	Firmicutes (f__Lachnospira)
	10-1	OTU00006	99.6	Firmicutes (*Clostridium perfringens*)
	10-2	OTU00032	100	Firmicutes (*Clostridium perfringens*)
	10-4	OTU00006	99.2	Firmicutes (*Clostridium perfringens*)
	11-2	OTU00688	100	Bacteroidetes (*Bacteroides fragilis*)
	11-4	OTU00006	100	Firmicutes (*Clostridium perfringens*)
	11-6	OTU00006	100	Firmicutes (*Clostridium perfringens*)
	11-7	OTU00132	99.6	Bacteroidetes (*Bacteroides fragilis*)
	12-1	OTU00125	99.6	Firmicutes (f__Peptostreptococcaceae)
	12-2	OTU00125	100	Firmicutes (f__Peptostreptococcaceae)
	12-3	OTU00108	100	Firmicutes (g__Enterococcus)
	12-4	OTU00108	99.6	Firmicutes (g__Enterococcus)
	12-5	OTU00125	100	Firmicutes (f__Peptostreptococcaceae)
	13-3	OTU00221	100	Firmicutes (f__Lachnospira)
	13-4	OTU00004	98.6	Firmicutes (f__Peptostreptococcaceae)
	2-1	OTU00938	99.8	Firmicutes (g__Oscillospira)
	2-2	OTU00321	98.8	Firmicutes (g__Oscillospira)
	26-1	OTU00050	98.8	Firmicutes (*Eubacterium dolichum*)
	26-10	OTU00006	100	Firmicutes (*Clostridium perfringens*)
	26-11	OTU00050	99.2	Firmicutes (*Eubacterium dolichum*)
	26-2	OTU00050	99.2	Firmicutes (*Eubacterium dolichum*)
	26-3	OTU00006	100	Firmicutes (*Clostridium perfringens*)
	26-4	OTU00050	99.2	Firmicutes (*Eubacterium dolichum*)
	26-5	OTU00050	99.2	Firmicutes (*Eubacterium dolichum*)
	26-6	OTU00006	100	Firmicutes (*Clostridium perfringens*)

26-7	OTU00050	99.2	Firmicutes (*Eubacterium dolichum*)
26-8	OTU00781	99.3	Firmicutes (*Eubacterium dolichum*)
26-9	OTU00050	99.2	Firmicutes (*Eubacterium dolichum*)
27-3	Otu00006	100	Firmicutes (*Clostridium perfringens*)
27-4	Otu00006	100	Firmicutes (*Clostridium perfringens*)
27-7	Otu00006	100	Firmicutes (*Clostridium perfringens*)
27-8	Otu00650	99.2	Firmicutes (f_Lachnospiraceae_unclassified)
27-9	Otu00016	99.6	Proteobacteria (f_Enterobacteriaceae_unclassified)
3-3	OTU00006	99.6	Firmicutes (*Clostridium perfringens*)
3-5	OTU00006	100	Firmicutes (*Clostridium perfringens*)
3-5 (2)	OTU00006	99.6	Firmicutes (*Clostridium perfringens*)
4-1	OTU00006	100	Firmicutes (*Clostridium perfringens*)
4-3	OTU00006	100	Firmicutes (*Clostridium perfringens*)
4-4	OTU00006	99.2	Firmicutes (*Clostridium perfringens*)
5-1	OTU00004	99.6	Firmicutes (f__Peptostreptococcaceae)
6-1	OTU00108	98.6	Firmicutes (g__Enterococcus)
6-2	OTU00108	100	Firmicutes (g__Enterococcus)
6-3	OTU00108	99.2	Firmicutes (g__Enterococcus)
6-4	OTU00108	100	Firmicutes (g__Enterococcus)
6-5	OTU00004	99.6	Firmicutes (f__Peptostreptococcaceae)
6-6	OTU00004	100	Firmicutes (f__Peptostreptococcaceae)
7-1	OTU00004	100	Firmicutes (f__Peptostreptococcaceae)
7-2	OTU00139	100	Bacteroidetes (*Parabacteroides distasonis*)
7-3	OTU00688	100	Bacteroidetes (*Bacteroides fragilis*)
7-4	OTU00139	100	Bacteroidetes (*Parabacteroides distasonis*)
7-6 (2)	OTU00139	100	Bacteroidetes (*Parabacteroides distasonis*)
7-6 (3)	OTU00108	99.6	Firmicutes (g__Enterococcus)
8-1	OTU00082	99.6	Firmicutes (g__SMB53(98))
8-2	OTU00006	100	Firmicutes (*Clostridium perfringens*)
8-3	OTU00061	100	Firmicutes (g__Pseudoramibacter_Eubacterium)
8-4	OTU00688	100	Bacteroidetes (*Bacteroides fragilis*)
8-5	OTU00004	98.8	Firmicutes (f__Peptostreptococcaceae)
8-5 (2)	OTU00004	98.4	Firmicutes (f__Peptostreptococcaceae)
8-6	OTU00082	99.6	Firmicutes (g__SMB53(98))
9-1	OTU00125	99.6	Firmicutes (f__Peptostreptococcaceae)
B26-1	OTU00688	100	Bacteroidetes (*Bacteroides fragilis*)
B26-2	OTU00688	100	Bacteroidetes (*Bacteroides fragilis*)
B26-3	OTU00166	100	Firmicutes (f__Peptostreptococcaceae)
B26-4	OTU00047	100	Firmicutes (g__Pseudoramibacter_Eubacterium)
B26-7	OTU00688	100	Bacteroidetes (*Bacteroides fragilis*)

	B26-8	OTU00047	100	Firmicutes (g__Pseudoramibacter_Eubacterium)
	B26-9	OTU00647	100	Bacteroidetes (g__Bacteroides)

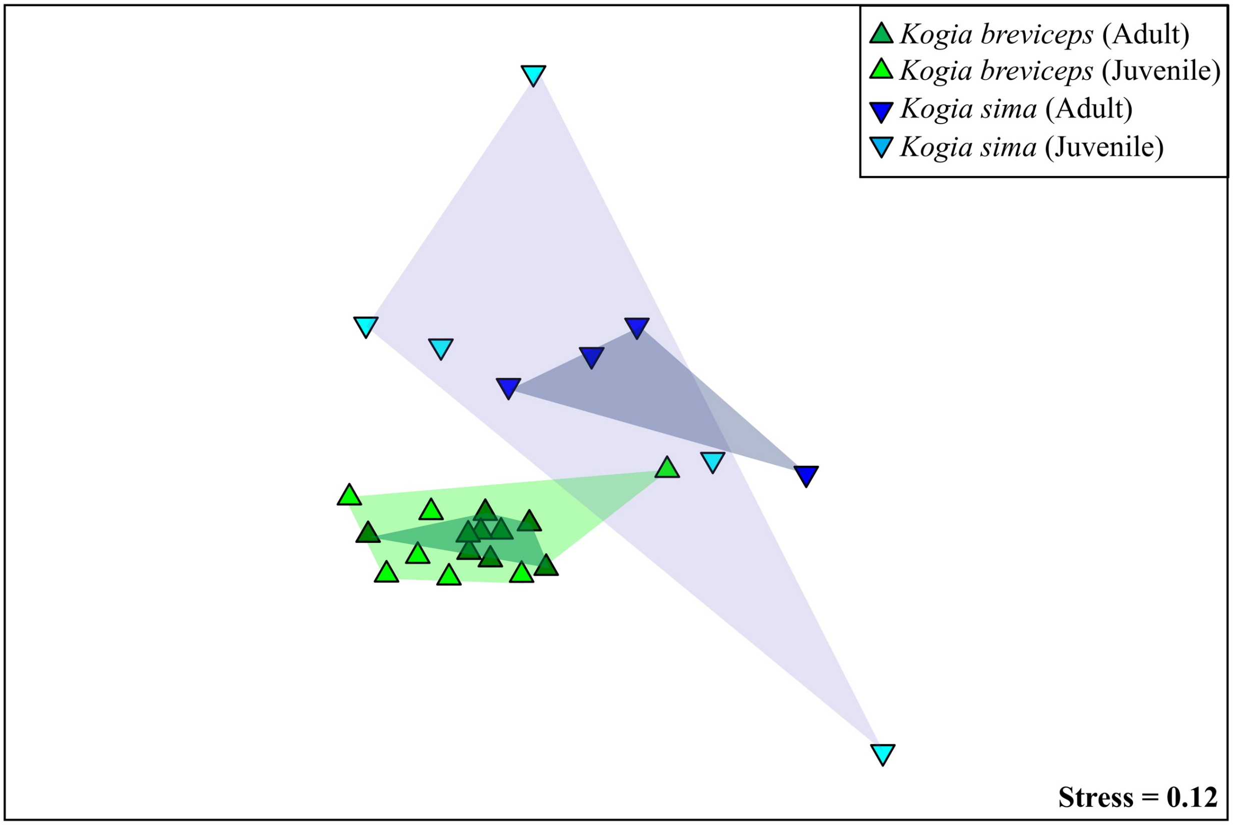

Figure S1. Non-metric multidimensional scaling (NMDS) plot of the gut microbiome in juvenile and adult *K. sima* (green shading) and *K. breviceps* (blue shading). Ordination is based on weighted UniFrac distance. Gut microbiomes differed significantly (PERMANOVA, $P<0.05$) across host species (*K. sima* vs. *K. breviceps*) and life stage (juvenile vs. adult).

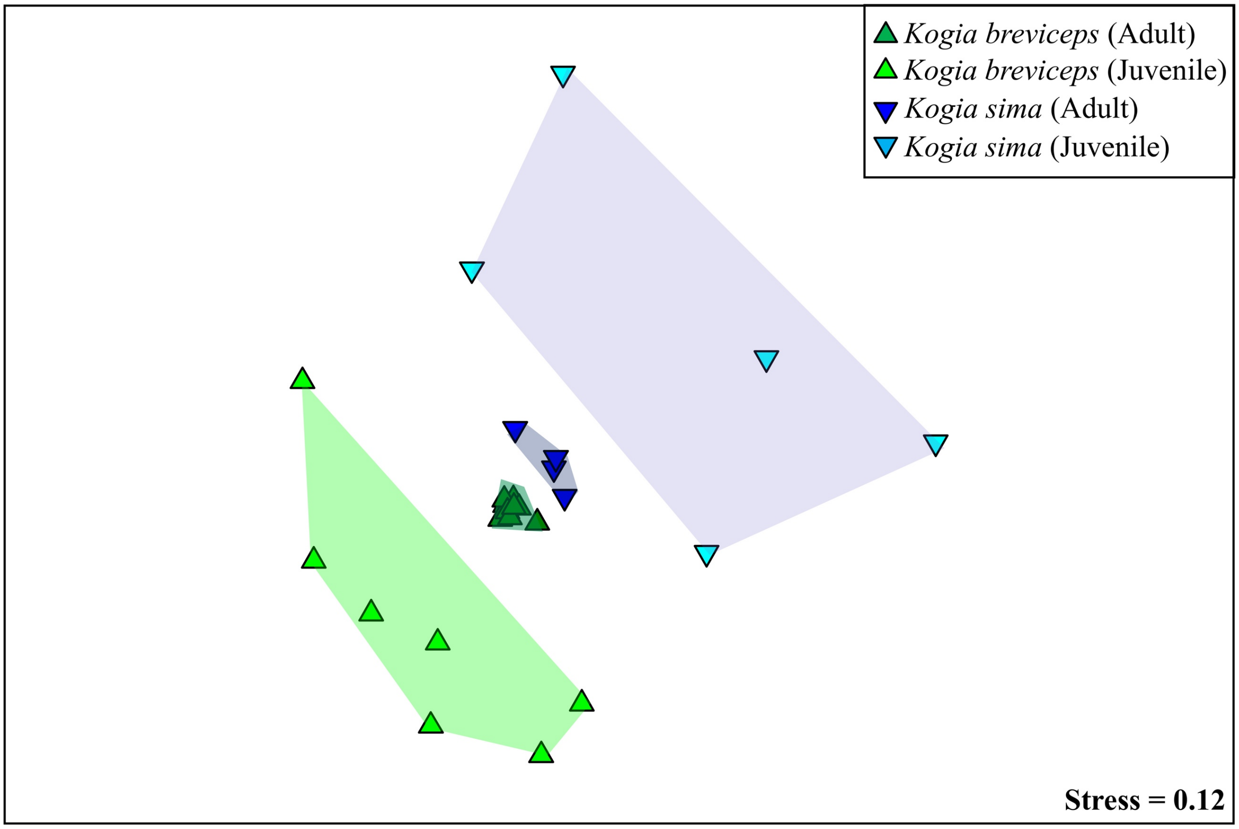

Figure S2. Non-metric multidimensional scaling (NMDS) plot of the gut microbiome in juvenile and adult *K. sima* (green shading) and *K. breviceps* (blue shading). Ordination is based on unweighted UniFrac distance. Gut microbiomes differed significantly (PERMANOVA, $P<0.05$) across host species (*K. sima* vs. *K. breviceps*) and life stage (juvenile vs. adult).

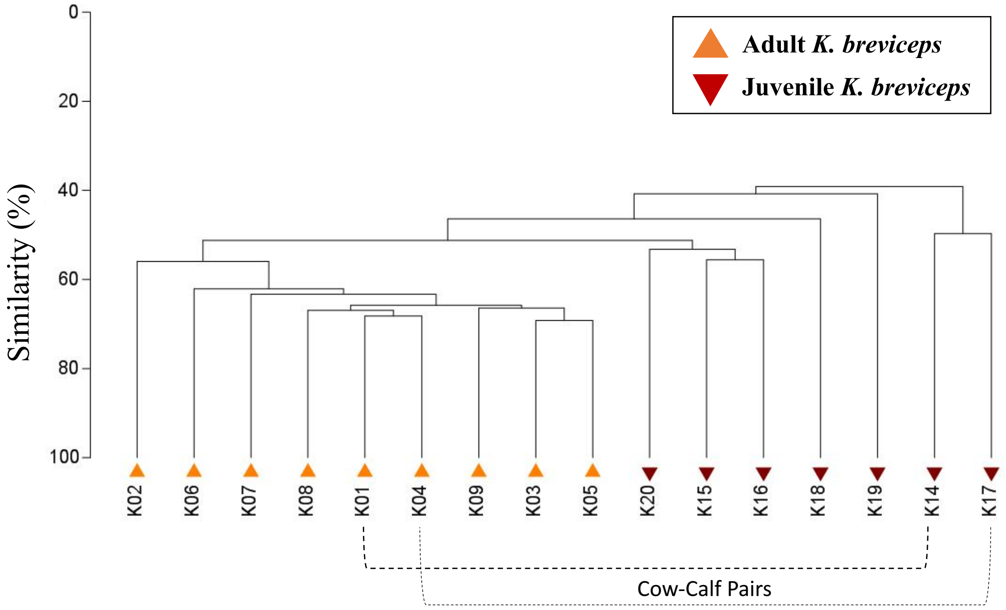

Figure S3. Gut microbiome similarity of cow-calf pairs (dotted line) compared to other juvenile and adult *K. breviceps* samples. Values represent microbiome similarity (Bray-Curtis) based on OTU relative abundances.
